# Aerial Swarm Defense by StringNet Herding: Theory and Experiments

**DOI:** 10.3389/frobt.2021.640446

**Published:** 2021-04-20

**Authors:** Vishnu S. Chipade, Venkata Sai Aditya Marella, Dimitra Panagou

**Affiliations:** ^1^Department of Aerospace Engineering, University of Michigan, Ann Arbor, MI, United States; ^2^Department of Electrical Engineering and Computer Science, University of Michigan, Ann Arbor, MI, United States

**Keywords:** decision making, swarm defense, autonomous robots and drones, cooperative control feedback, multi-agent system

## Abstract

This paper studies a defense approach against one or more swarms of adversarial agents. In our earlier work, we employed a closed formation (“StringNet”) of defending agents (defenders) around a swarm of adversarial agents (attackers) to confine their motion within given bounds, and guide them to a safe area. The adversarial agents were assumed to remain close enough to each other, i.e., within a prescribed connectivity region. To handle situations when the attackers no longer stay within such a connectivity region, but rather split into smaller swarms (clusters) to maximize the chance or impact of attack, this paper proposes an approach to learn the attacking sub-swarms and reassign defenders toward the attackers. We use a “Density-based Spatial Clustering of Application with Noise (DBSCAN)” algorithm to identify the spatially distributed swarms of the attackers. Then, the defenders are assigned to each identified swarm of attackers by solving a constrained generalized assignment problem. We also provide conditions under which defenders can successfully herd all the attackers. The efficacy of the approach is demonstrated via computer simulations, as well as hardware experiments with a fleet of quadrotors.

## 1. Introduction

Rapid advancements in swarm technology and its increasing presence in airspace pose significant threat to safety-critical infrastructure such as government facilities, airports, and military bases. The presence of adversarial swarms nearby such entities, with the aim of causing physical damage or collecting critical information, can lead to catastrophic consequences. This necessitates solutions for the protection of safety-critical infrastructure against such attacks, particularly in populated areas.

Counteracting an adversarial swarm by means of physical interception, as studied in Chen et al. (2017), Coon and Panagou (2017), and Shishika et al. (2020), may not be desirable at low altitudes in an urban environment due to human presence. Under the assumption of risk-averse and self-interested adversarial agents (attackers) that tend to move away from the defending agents (defenders) and from other dynamic objects, *herding* can be used as an indirect way of guiding the attackers to some safe area in order to safe-guard a safety-critical area (protected area).

In our recent work Chipade and Panagou (2019, 2020b), we developed a herding algorithm, called “StringNet Herding,” to herd a swarm of attackers away from a protected area. A closed formation (“StringNet”) of defending agents connected by string barriers is formed around a swarm of attackers to confine their motion within given bounds, and guide them to a safe area. However, the assumption that the attackers stay together in a circular region, and that they react to the defenders collectively as a single swarm while attacking the protected area, can be quite conservative in practice.

In this paper, we build upon our earlier work on “StringNet Herding” (Chipade and Panagou, 2020b) and study the problem of defending a protected area from attackers that *may* or *may not* stay together throughout their attack. We propose a “Multi-Swarm StringNet Herding” approach that uses clustering-based defender assignment, and the “StringNet Herding” method to herd the multiple swarms adversarial attackers' to known safe areas.

### 1.1. Related Work

Herding has been studied earlier in the literature, see for instance (Haque et al., 2011; Paranjape et al., 2018; Pierson and Schwager, 2018). The approach in Paranjape et al. (2018) uses an *n*-wavefront algorithm to herd a flock of birds away from an airport, where the birds on the boundary of the flock are influenced based on the locations of the airport and a safe area.

The herding method in Pierson and Schwager (2018) utilizes a circular-arc formation of herders to influence the non-linear dynamics of the herd based on a potential-field approach, and designs a point-offset controller to guide the herd close to a specified location. In Haque et al. (2011), biologically-inspired strategies are developed for confining a group of agents; the authors develop strategies based on the “wall” and “encirclement” methods that dolphins use to capture a school of fish. In addition, they compute regions from which this confinement is possible; however, the results are limited to constant-velocity motion. A similar approach called *herding by caging* is adopted in Varava et al. (2017), where a cage of high potential is formed around the attackers. An RRT approach is used to find a motion plan for the agents; however, the cage is assumed to have already been formed around the agents, while the caging of the agents thereafter is only ensured with constant velocity motion under additional assumptions on the distances between the agents. Forming such a cage could be more challenging in case of self-interested, risk-averse attackers under non-constant velocity motion.

In Licitra et al. (2017, 2018), the authors discuss herding using a switched-system approach; the herder (defender) chases targets (evaders/attackers) sequentially by switching among them so that certain dwell-time conditions are satisfied to guarantee stability of the resulting trajectories. However, the assumption that only one of the targets is influenced by the herder at any time might be limiting and non-practical in real applications. The authors in Deptula et al. (2018) use approximate dynamic programming to obtain suboptimal control policies for the herder to chase a target agent to a goal location. A game-theoretic formulation is used in Nardi et al. (2018) to address the herding problem by constructing a virtual barrier similar to Pierson and Schwager (2018). However, the computational complexity due to the discretization of the state and control-action spaces limits its applicability.

Most of the aforementioned approaches for herding are limiting due to one or some of the following aspects: (1) simplified motion models (Varava et al., 2017; Pierson and Schwager, 2018), (2) absence of obstacles in the environment (Licitra et al., 2017, 2018; Paranjape et al., 2018), (3) no consideration of intra-team collisions (Varava et al., 2017; Pierson and Schwager, 2018), (4) assumption on a particular form of potential field to model the repulsive motion of the attackers with respect to the defenders (Licitra et al., 2017, 2018; Paranjape et al., 2018; Pierson and Schwager, 2018).

We have addressed the above issues in our recent work Chipade and Panagou (2019, 2020b), which develops a method termed as “StringNet Herding,” for defending a protected area from a swarm of attackers in a 2D obstacle environment. In “StringNet Herding,” a closed formation of strings (“StringNet”) is formed by the defenders to surround the swarm of attackers. It is assumed that the attackers will stay together within a circular footprint as a swarm and collectively avoid the defenders. It is also assumed that the string between two defenders serves as a barrier through which the attackers cannot escape (e.g., a physical straight-line barrier, or some other mechanism). The StringNet is then controlled to herd the swarm of attackers to a safe area. The control strategy for the defenders in “StringNet Herding” is a combination of time-optimal control actions and finite-time, state-feedback, bounded control actions, so that the attackers can be herded to safe area in a timely manner.

Clustering of data points is a popular machine learning technique (Xu and Tian, 2015). There are various types of clustering algorithms: partition based (K-means; MacQueen et al., 1967), hierarchy based (BIRCH; Zhang et al., 1996), density based (DBSCAN; Ester et al., 1996), stream based (STREAM; O'Callaghan et al., 2002), graph based (CLICK; Sharan and Shamir, 2000). In the context of dynamical systems, authors in Cai et al. (2017) develop a clustering method based on quasi-consensus motions of dynamic agents where agents belonging to a particular cluster are expected to aggregate together. This method however converges to clustering results asymptotically. In this paper, we are interested in spatial proximity of the agents during a finite future time. So, we focus mostly on the density based approaches, for example, DBSCAN, to solve the clustering problem in this paper.

Assignment problems have also been studied extensively (Burkard et al., 2012). A detailed survey of an assignment problem pertaining to defense scenarios called “weapon-target assignment (WTA)” problem is provided in Kline et al. (2019). In general, assignment problems are NP-hard, and hence can be solved only approximately for large number of decision variables. For example, authors in Rezende et al. (2018) provide a greedy approach based on ant colony system to solve the WTA problem. Multi-agent defense problems are difficult to solve optimally because the problem becomes computationally intractable for large number of agents. In such cases, all possible pairwise games are first solved, and then an assignment problem is solved to assign defending agents against attacking ones based on the cost of the pairwise games. For example in Chen et al. (2017) and Coon and Panagou (2017), after solving the pairwise games, the defenders are assigned to attackers by solving a bipartite matching problem using Hungarian algorithm (Kuhn, 1955). Similarly, in Yan et al. (2019) authors solve a mixed integer program to find assignment of defenders to attackers in a multiplayer reach-avoid game played in a convex domain. In this paper, we are interested in a generalized assignment problem (GAP) (Öncan, 2007), in which there are more number of objects than knapsacks to be filled, because we aim to assign groups of defenders to a number of attackers' clusters, which are typically small in number than the number of defenders. Similar to the standard assignment problems, GAP is known to be NP-hard, but there are approximation algorithms to solve an arbitrary instance of GAP (Öncan, 2007).

### 1.2. Overview of the Proposed Approach

In the preliminary work presented in Chipade and Panagou (2020c), we extended the “StringNet Herding” approach to scenarios where attackers no longer stay together and may split into smaller swarms in reaction to the defenders' presence. The proposed approach involves: (1) identification of the clusters (swarms) of the attackers that stay together, (2) distribution and assignment of the defenders to each of the identified swarms of the attackers, (3) use of “StringNet Herding” approach by the defenders to herd each identified swarm of attackers to the closest safe area.

More specifically, we use the “Density based Spatial Clustering of Application with Noise (DBSCAN)" algorithm (Ester et al., 1996) to identify swarms of the attackers based on the proximity of the attackers to each other. We then formulate a generalized assignment problem with additional constraints on the connectivity of the defenders, to find which defender should go against which swarm of attackers and herd it to one of the safe areas. This connectivity constrained generalized assignment problem (C2GAP) is modeled as a mixed integer quadratically constrained program (MIQCP) to obtain an optimal assignment solution. Additionally, we provide a hierarchical algorithm to find the assignment quickly.

In this paper, we further improve the clustering based multi-swarm herding approach by developing a decentralized variant of the MIQCP that is used to assign the defenders to the identified swarms of the attackers. Furthermore, we address the question of whether the defenders starting at some known positions can gather on the shortest path of the attackers, starting at some states, to the protected area and can successfully herd the attackers to safe areas. We also demonstrate the “StringNet Herding” algorithm for single attacking swarm case via hardware experiments with a fleet of quadrotor vehicles that are capable of flying autonomously in an outdoor aerial robotics facility at the University of Michigan campus.

### 1.3. Summary of our Contributions

In summary, compared to the prior literature and our prior work presented in the conference version (Chipade and Panagou, 2020c), the novelties and contribution of this work are:

- a decentralized cooperative algorithm to group and assign the defenders to herd the identified different swarms of the attackers to the closest safe areas;
- a set of conditions under which the defenders are able to gather on the shortest path of the oncoming attackers to the protected area before the attackers could reach the gathering location and thereafter herd all the attackers to the safe areas;
- a demonstration of the “StringNet Herding” approach for a single attacking swarm case in hardware experiments using a fleet of quadrotor vehicles equipped with autonomous flight capability.

### 1.4. Structure of the Paper

Section 2 describes the mathematical modeling and problem statement. The “StringNet Herding” approach to herd a single swarm of attackers is briefly discussed in section 3. The approach on identification of attackers' swarms (clusters) and the defenders' assignment to these identified swarms for multiple-swarm herding is discussed in section 4. Simulations are provided in section 7, and the hardware experiments are discussed in section 8. Finally, the paper is concluded in section 10.

## 2. Modeling and Problem Statement

*Notation*: We use **r**, **v** and **u** to denote position, velocity and input acceleration vector, respectively. We use ***ξ*** and ***η*** to denote desired position and velocity vector, respectively. We use *A* and *D* to denote the indices of attackers and defenders, respectively, while *I* denotes order set of positive integers starting at 1. The variables *ai, ac*_*k*_, *dj, dc*_*k*_ used as subscripts of the above variables correspond to the *i*^*th*^ attacker, center of mass of *k*^*th*^ swarm of attackers, *j*^*th*^ defender and the *k*^*th*^ group of defenders, respectively. Similarly, subscripts *p*, *sm* denote the protected area and *m*^*th*^ safe area, respectively. We use subscript *d* to denote common variables that correspond to all the defenders and subscripts *a* to denote common variables corresponding to all the attackers. We use *sn* and *sb* as a subscripts to denote StringNet and string barrier, respectively. Any variable with superscript *g*, *s*, *e*, *h* correspond to gathering, seeking, enclosing and herding phase, respectively.

The set of integers greater than 0 is denoted by ℤ_>0_. ‖.‖ denotes the Euclidean norm of its argument. |.| denotes the absolute value of a scalar, and cardinality if the argument is a set. ⌊·⌋ gives the largest integer smaller than the argument number. A ball of radius ρ centered at the origin is defined as $B_{\rho }=\left\{\text{r}\in {\mathbb{R}}^{2}|\left\|\text{r}\right\|\leq \rho \right\}$.

We consider *N*_*a*_ attackers $A_{i}$, *i* ∈ *I*_*a*_ = {1, 2, …, *N*_*a*_}, and *N*_*d*_ defenders $D_{j}$, *j* ∈ *I*_*d*_ = {1, 2, …, *N*_*d*_}, operating in a 2D environment $W\subseteq {\mathbb{R}}^{2}$ that contains a protected area P⊂W, defined as $P=\left\{\text{r}\in {\mathbb{R}}^{2}|\left\|\text{r}-{\text{r}}_{p}\right\|\leq \rho_{p}\right\}$, and *N*_*s*_ safe areas $S_{m}\subset W$, defined as $S_{m}=\left\{\text{r}\in {\mathbb{R}}^{2}|\left\|\text{r}-{\text{r}}_{sm}\right\|\leq \rho_{sm}\right\}$, for all *m* ∈ *I*_*s*_ = {1, 2, …, *N*_*s*_}, where (**r**_*p*_, ρ_*p*_) and (**r**_*sm*_, ρ_*sm*_) are the centers and radii of the corresponding areas, respectively. The attackers aim to reach the protected area P. The attackers may use flocking controllers (Dai and Li, 2014) to stay together, or they may choose to split into different smaller swarms (Raghuwaiya et al., 2016; Goel et al., 2019). The defenders aim to herd each of these attackers to one of the safe areas in $S=\left\{S_{1},S_{2},\ldots ,S_{N_{s}}\right\}$ before they reach P.

The agents $A_{i}$ and $D_{j}$ are modeled as discs of radii ρ_*a*_ and ρ_*d*_(≤ ρ_*a*_), respectively and move under double integrator (DI) dynamics with quadratic drag (damped double integrator):

(1) $$\begin{array}{l} \begin{array}{c} {\dot{\text{r}}}_{ai}={\text{v}}_{ai},\;{\dot{\text{v}}}_{ai}={\text{u}}_{ai}-C_{D}{\|\text{v}}_{ai}\|{\text{v}}_{ai}; \end{array} \end{array}$$

(2) $$\begin{array}{l} \begin{array}{c} {\dot{\text{r}}}_{dj}={\text{v}}_{dj},\;{\dot{\text{v}}}_{dj}={\text{u}}_{dj}-C_{D}{\|\text{v}}_{dj}\|{\text{v}}_{dj}; \end{array} \end{array}$$

(3) $$\begin{array}{l} \begin{array}{c} {\left\|\text{u}\right\|}_{ai}\leq {\bar{u}}_{a},\;{\|\text{u}}_{dj}\|\leq {\bar{u}}_{d}, \end{array} \end{array}$$

where *C*_*D*_ is the drag coefficient, ${\text{r}}_{ai}={\left[x_{ai},y_{ai}\right]}^{T}$ and ${\text{r}}_{dj}={\left[x_{dj},y_{dj}\right]}^{T}$ are the position vectors of $A_{i}$ and $D_{j}$, respectively; ${\text{v}}_{ai}={\left[v_{x_{ai}},v_{y_{ai}}\right]}^{T}$, ${\text{v}}_{dj}={\left[v_{x_{dj}},v_{y_{dj}}\right]}^{T}$ are the velocity vectors, respectively, and ${\text{u}}_{ai}={\left[u_{x_{ai}},u_{y_{ai}}\right]}^{T}$, ${\text{u}}_{dj}={\left[u_{x_{dj}},u_{y_{dj}}\right]}^{T}$ are the accelerations (the control inputs), respectively. This model poses a speed bound on each player with limited acceleration control, i.e., $v_{ai}=\left\|{\text{v}}_{ai}\right\|<{\overset{-}{v}}_{a}=\sqrt{\frac{{\bar{u}}_{a}}{C_{d}}}$ and $v_{dj}=\left\|{\text{v}}_{dj}\right\|<{\overset{-}{v}}_{d}=\sqrt{\frac{{\bar{u}}_{d}}{C_{d}}}$. The defenders are assumed to be faster than the attackers, i.e., ${\overset{-}{v}}_{a}<{\overset{-}{v}}_{d}$ (i.e., ū_*a*_ < ū_*d*_). The number of defenders is assumed to be no less than that of attackers, i.e., *N*_*d*_ ≥ *N*_*a*_.

**Figure 1:**
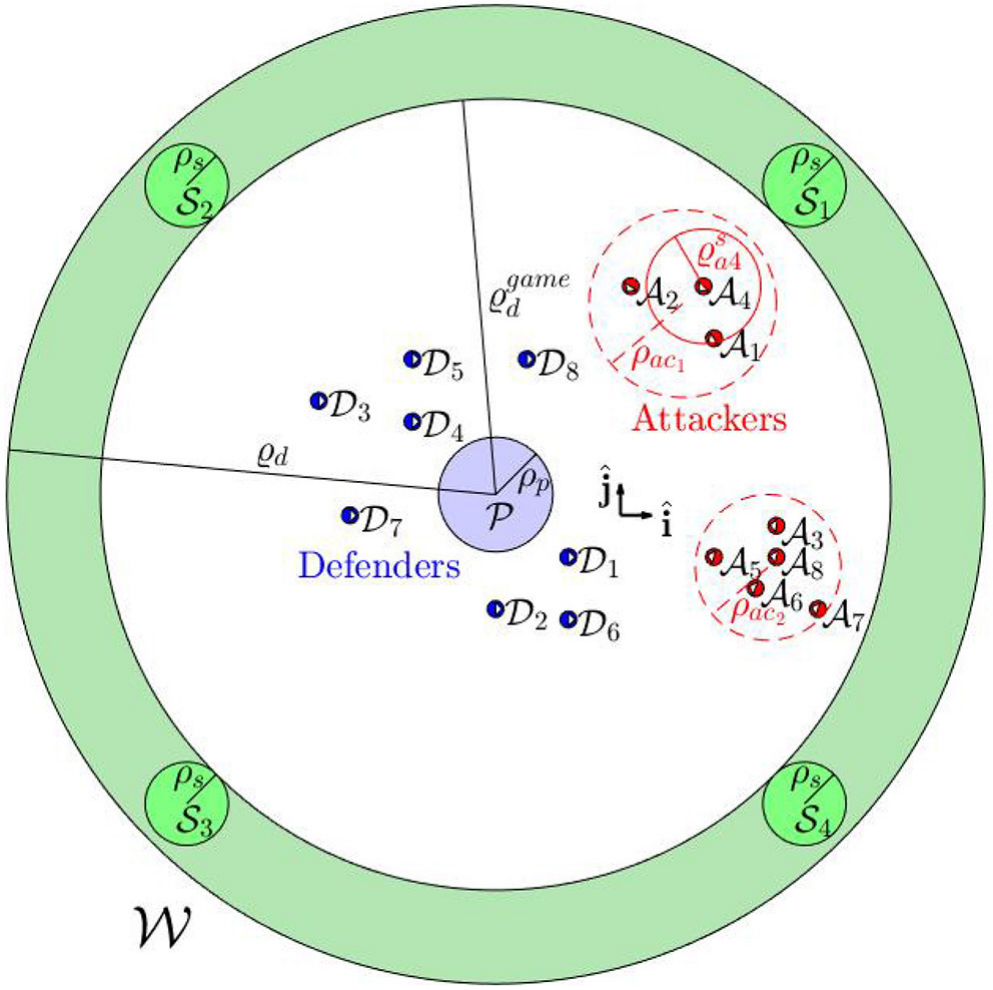
Problem formulation.

There is a distributed navigation system that senses the position **r**_*ai*_ and velocity **v**_*ai*_ of the attacker $A_{i}$ that lies inside a circular sensing zone Zd={r∈ℝ2|‖r-rpa‖≤ϱd} for all *i* ∈ *I*_*a*_, where ϱ_*d*_ > 0 is the radius of the defenders' sensing zone. The navigation system communicates the sensed information to the defenders $D_{j}$, for all *j* ∈ *I*_*d*_. Every attacker $A_{i}$ has a local sensing zone Zai={r∈ℝ2|‖r-rai‖≤ϱai}, where ϱ_*ai*_ > 0 is the radius of the attacker $A_{i}$'s sensing zone (Figure 1). This navigation system can include sensors such as radars, lidars, cameras that are spatially distributed around the protected area and provide measurements of positions and velocities of the attackers and the defenders. The defenders are also assumed to have sufficient computational power available on board to solve the assignment problems that are discussed later in the paper.

Formally, we consider the followin g problems.

**Problem 1** (Swarm Identification). *Identify the swarms* $\left\{A_{c_{1}},A_{c_{2}},\ldots ,A_{c_{N_{ac}}}\right\}$ *of the attackers for some unknown *N*_*ac*_ ≥ 1 such that attackers in the same swarm* $A_{c_{k}}$, *and only them, are physically close to each other and satisfy prescribed conditions (described later) on spatial density, where* $A_{c_{k}}=\left\{A_{i}|i\in A_{c_{k}}\right\}$, *A*_*c*_*k*__ ⊆ *I*_*a*_, *for all* *k* ∈ *I*_*ac*_ = {1, 2, …, *N*_*ac*_}.

**Problem 2** (Multi-Swarm Herding). *Find subgroups* $\left\{D_{c_{1}},D_{c_{2}},\ldots ,D_{c_{N_{ac}}}\right\}$ *of the defenders and their assignment to the attackers' swarms identified in Problem 1, such that all the defenders in the same subgroup are connected via string barriers to enclose and herd the assigned attacker's swarm.*

**Problem 3** (Defenders' Dominance Region). *Given the initial positions of the defenders* **r**_*dj*_(0), *for all *j* ∈ *I*_*d*_, provide conditions on the initial positions* **r**_*ai*_(0), *for all *i* ∈ *I*_*a*_, of the attackers for which the defenders are able to gather as a specified formation centered at a point on the expected path of the attackers before any attacker reaches the center of the formation*.

Before we discuss the solutions to the above three problems, we first briefly describe the “StringNet Herding” approach used to herd a single swarm of the attackers in the following section.

## 3. Herding a Single Swarm of Attackers

To herd a swarm of attackers to S, we use “StringNet Herding,” developed in Chipade and Panagou (2020b). StringNet is a closed net of strings formed by the defenders as shown in **Figure 3**. The strings are realized as impenetrable and extendable line barriers (e.g., spring-loaded pulley and a rope or other similar mechanism; Mirjan et al., 2016) that prevent attackers from passing through them. The extendable string barrier allows free relative motion of the two defenders connected by the string. The string barrier can have a maximum length of ${\overset{-}{R}}_{sb}>0$. If the string barrier were to be physical one, then it can be established between two defenders $D_{j}$ and $D_{j^{\prime }}$ only when they are close to each other and have almost same velocity, i.e., $\left\|{\text{r}}_{dj}-{\text{r}}_{dj^{\prime }}\right\|\leq \epsilon_{1}<{\overset{-}{R}}_{sb}$ and $\left\|{\text{v}}_{dj}-{\text{v}}_{dj^{\prime }}\right\|\leq \epsilon_{2}$, where ϵ_1_ and ϵ_2_ are small numbers. The underlying graph structure for the two different “StringNet” formations defined for a subset of defenders $D^{\prime }=\left\{D_{j}|j\in I_{d}^{\prime }\right\}$, where $I_{d}^{\prime }\subseteq I_{d}$, are defined as follows:

**Definition 1** (Closed-StringNet). *The Closed-StringNet* $G_{sn}^{cl}\left(I_{d}^{\prime }\right)=(V_{sn}^{cl}\left(I_{d}^{\prime }\right),$ $E_{sn}^{cl}\left(I_{d}^{\prime }\right))$ *is a cycle graph consisting of: 1) a subset of defenders as the vertices*, $V_{sn}^{cl}\left(I_{d}^{\prime }\right)=\left\{D_{j}|j\in I_{d}^{\prime }\right\}$, *2) a set of edges*, $E_{sn}^{cl}\left(I_{d}^{\prime }\right)=\left\{\left(D_{j},D_{j^{\prime }}\right)\in V_{sn}^{cl}\left(I_{d}^{\prime }\right)\times V_{sn}^{cl}\left(I_{d}^{\prime }\right)|D_{j}\overset{s}{↔}D_{j^{\prime }}\right\}$, *where the operator* $\overset{s}{↔}$ *denotes an impenetrable line barrier between the defenders*.

**Definition 2** (Open-StringNet). *The Open-StringNet* $G_{sn}^{op}\left(I_{d}^{\prime }\right)=(V_{sn}^{op}\left(I_{d}^{\prime }\right),$ $E_{sn}^{op}\left(I_{d}^{\prime }\right))$ *is a path graph consisting of: 1) a set of vertices*, $V_{sn}^{op}\left(I_{d}^{\prime }\right)$ *and 2) a set of edges,* $E_{sn}^{op}\left(I_{d}^{\prime }\right)$*, similar to that in Definition 1*.

**Figure 2:**
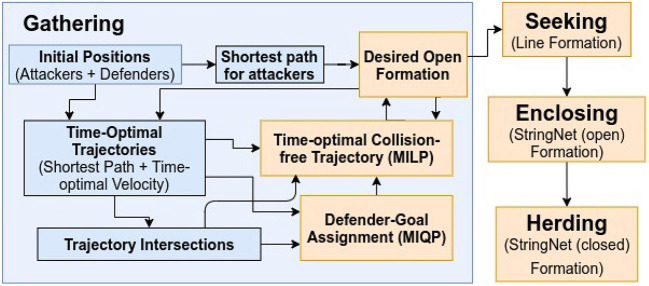
Block diagram for “StringNet Herding”.

The StringNet herding consists of four phases: (1) gathering, (2) seeking,(3) enclosing, and (4) herding to a safe area, see the block diagram in Figure 2.

These phases are discussed as follows.

### 3.1. Gathering

**Figure 3:**
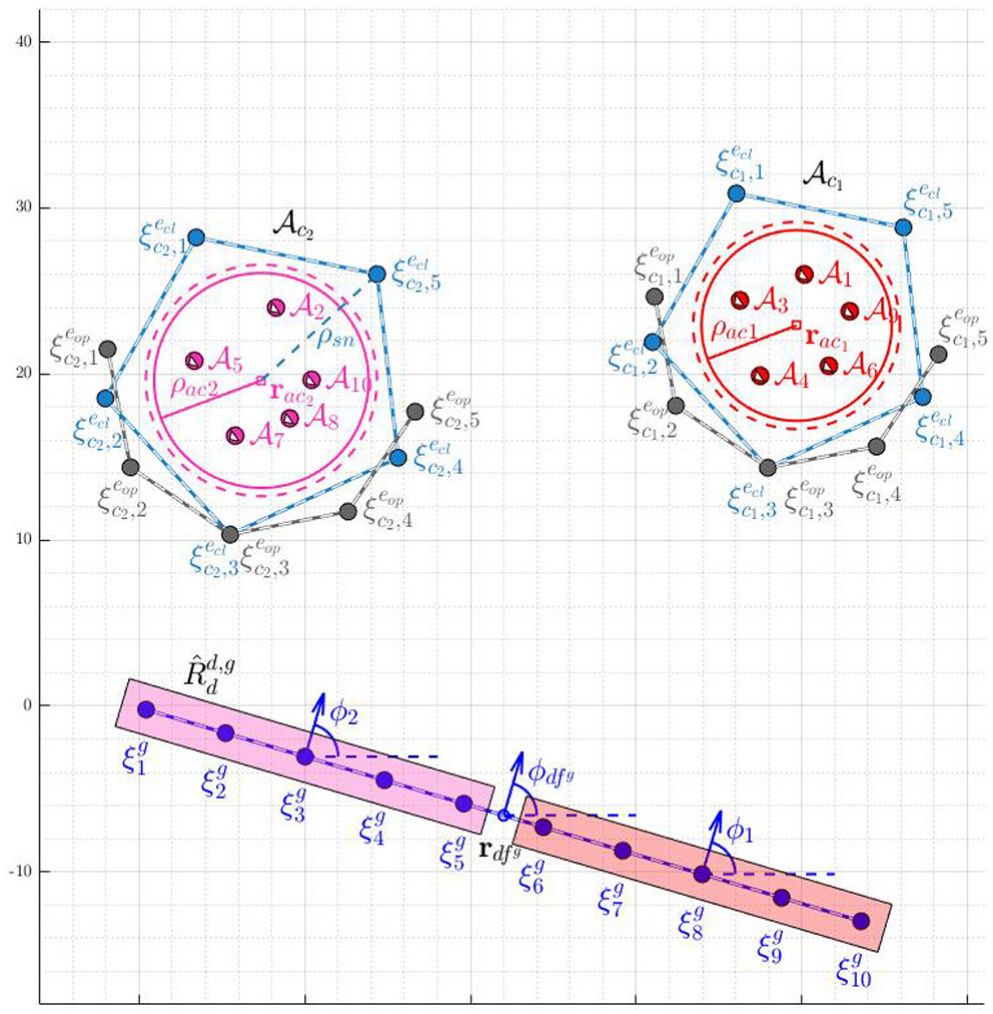
Assignment of defenders to the attackers' swarms.

We assume that the attackers start as single swarm that stays together and they may start splitting into smaller groups as they sense the defenders in their path. The aim of the defenders is to converge to an open formation 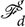 centered at the gathering center ${\text{r}}_{dc^{g}}$ located on the expected path of the attackers, where the expected path is defined as the shortest path of the attackers to the protected area, before the attackers reach ${\text{r}}_{dc^{g}}$ (see Figure 3). Let 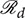
(*N*_*a*_):ℤ_>0_ → ℤ_>0_ be the resource allocation function that outputs the number of the defenders that can be assigned to the given *N*_*a*_ attackers. We make the following assumption about the resource allocation function.

**Assumption 1**. *The resource allocation function is a strictly monotonically increasing function, i.e.,* 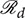
*N*_*a*_) < 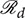
(*N*_*a*_ + 1) *and satisfies* 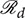
(*N*_*a*_) ≥ *N*_*a*_.

Assumption 1 ensures that there are adequate number of defenders to go after each attacker in the event the attackers in the swarm disintegrate into singular swarms (swarms with less than 3 attackers). In the case of large number of singular swarms, herding may not be the most economical way of defense as there needs to be at least 3 defenders to form a Closed-StringNet. In which case, one could employ a different mechanism to counteract the attack, for example, physical capture or interception of the attacker. In this paper we only the consider the attackers' swarms with greater than or equal to 3 attackers. The case of singular swarms will be studied in our future work.

The open formation 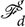 is characterized by the positions $\xi_{l}^{g}$, for all *l* ∈ *I*_*d*_*c*__0__ = {1, 2, …, 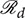
(*N*_*a*_)}, and is chosen to be a straight line formation^1^ (see Figure 3). Once the defenders arrive at these positions, the defenders get connected by strings as follows: the defender at $\xi_{l}^{g}$ gets connected to the defender at $\xi_{l+1}^{g}$ for all 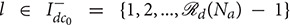 (see Figure 3). The angle made by the normal to the line joining $\xi_{1}^{g}$ and $\xi_{N_{d}}^{g}$ (clockwise from $\xi_{1}^{g}$, see Figure 3) is the orientation ϕ of the formation. The formation 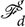 is chosen such that its orientation is toward the attackers on their expected path (defined above), see the blue formation in Figure 3. The desired positions $\xi_{l}^{g}$ on 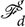 centered at ${\text{r}}_{dc^{g}}$ are:

(4) $$\begin{array}{l} \begin{array}{c} \xi_{l}^{g}={\text{r}}_{dc^{g}}+R_{l}\hat{\text{o}}\left(\theta_{dc^{g}}+\frac{\pi }{2}\right),\text{ for all }l\in I_{dc_{0}}; \end{array} \end{array}$$

where 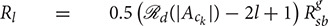, $\hat{\text{o}}\left(\theta \right)={\left[\cos\left(\theta \right),\sin\left(\theta \right)\right]}^{T}$ is the unit vector making an angle θ with *x*-axis, $\theta_{dc^{g}}=\theta_{a_{cm}}^{*}+\pi$, where $\theta_{a_{cm}}^{*}$ is the angle made by the line segment joining the attackers' center of mass (ACoM) to the center of the protected area (the shortest path from the initial position of ACoM to P) with *x*-axis. These positions are static, i.e., ${\dot{\xi }}_{l}^{g}={\ddot{\xi }}_{l}^{g}=\text{0}$. The gathering center ${\text{r}}_{dc^{g}}=\rho_{df}^{g}\hat{\text{o}}\left(\theta_{dc^{g}}\right)$ is such that $\rho_{df}^{g}>\rho_{p}$. We define the defender-goal assignment as:

**Definition 3** (Defender-Goal Assignment). *An injective mapping* β_0_:{1, 2, …, 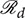
(*N*_*a*_)} → *I*_*d*_ *such that the defender* $D_{\beta_{0}\left(l\right)}$ *is assigned to go to the goal* $\xi_{l}^{g}$.

As discussed in Algorithm 1 in Chipade and Panagou (2020b) and as shown in Figure 2, we design a time-optimal motion plan so that the defenders gather at the desired formation 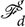 as early as possible and before the attackers reach close to 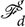. The idea in Algorithm 1 in Chipade and Panagou (2020b) is to iteratively solve a mixed integer quadratic program (MIQP) until a gathering center for the gathering formation is found which is as far as possible from the protected area and such that the defenders are able to gather at the formation 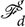 centered at the gathering center with bounded acceleration before the attackers can.

### 3.2. Seeking

After the defenders accomplish gathering, suppose a group of defenders $D_{c_{k}}=\left\{D_{j}|j\in D_{c_{k}}\right\}$, *D*_*c*_*k*__ ⊆ *I*_*d*_, is tasked to herd a swarm of attackers $A_{c_{k}}=\left\{A_{i}|i\in A_{c_{k}}\right\}$, *A*_*c*_*k*__ ⊆ *I*_*a*_, the details are discussed later in section 4. Denote $I_{dc_{k}}=\left\{1,2,\ldots ,|D_{c_{k}}|\right\}$ and let β_*k*_:*I*_*d*_*c*__*k*__ → *D*_*c*_*k*__ be the mapping that gives the indexing order of the defenders in $D_{c_{k}}$ on the Open-StringNet line formation 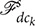 (similar to 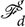 but with ${\overset{-}{R}}_{sb}$ being the distance of each defender from their immediate neighbor). In the seeking phase, the defenders in $D_{c_{k}}$ maintain the line formation 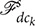 and try to get closer to the swarm of attackers $A_{c_{k}}$ by using state-feedback, finite-time convergent, bounded control laws as discussed in Chipade and Panagou (2020b). The control actions in Chipade and Panagou (2020b) for the defenders in $D_{c_{k}}$ are modified to incorporate collision avoidance from the other StringNet formations formed by $D_{c_{k^{\prime }}}$, for *k*′ ≠ *k*.

### 3.3. Enclosing: Closed-StringNet Formation

Once the Open-StringNet formation reaches close to the attackers' formation, the defenders start enclosing the attackers by moving to their desired positions on the enclosing formations while staying connected to their neighbors. We choose two formations for this phase that the defenders sequentially achieve: (1) Semi-circular Open-StringNet formation (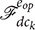), (2) Circular Closed-StringNet formation (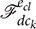). When the defenders directly try to converge to a circular formation from a line formation during this phase, the defenders at the either end of the Open-StringNet formation will start coming closer to each other reducing the length of the overall barrier in the attackers' path significantly. This is because the desired positions of these terminal defenders in the circular formation would be very close to each other on the opposite side of the circular formation (see Figure 3) and collision avoidance part of the controller is only active locally near the circle of maximum radius ${\overset{-}{\rho }}_{ac_{k}}$ around the swarm $A_{c_{k}}$. So the defenders would first converge to a semi-circular formation and would converge to a circular formation after the former is achieved.

The desired position $\xi_{c_{k},l}^{e_{op}}$ on the Open-StringNet formation 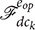 (Figure 3) is chosen on the circle with radius ρ_*sn*_*k*__ centered at **r**_*ac*_*k*__ as:

(5) $$\begin{array}{l} \begin{array}{c} \xi_{c_{k},l}^{e_{op}}={\text{r}}_{ac_{k}}+\rho_{sn_{k}}\hat{\text{o}}\left(\theta_{l}\right),\text{where }\theta_{l}=\theta_{dc_{k}}^{e*}+\frac{\pi }{2}+\frac{\pi \left(l-1\right)}{|D_{c_{k}}|-1}, \end{array} \end{array}$$

for all *l* ∈ *I*_*dc*_*k*__, where $\theta_{dc_{k}}^{e*}=\theta_{dc_{k}}^{s*}$. ${\text{r}}_{ac_{k}}=\underset{i\in I_{ac_{k}}}{\sum }\frac{{\text{r}}_{ai}}{\left|A_{c_{k}}\right|}$ is the center of mass of $A_{c_{k}}$. The radius ρ_*sn*_*k*__ should satisfy, ${\overset{-}{\rho }}_{ac_{k}}+b_{d}<\rho_{sn_{k}}$, where ${\overset{-}{\rho }}_{ac_{k}}$ is maximum radius of swarm $A_{c_{k}}$. The parameter *b*_*d*_ is the tracking error for the defenders in this phase (Chipade and Panagou, 2020b).

Similarly, the desired positions $\xi_{c_{k},l}^{e_{cl}}$ on the Closed-StringNet formation 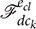 same as in Equation (5) with $\theta_{l}=\theta_{dc_{k}}^{e*}+\frac{\pi \left(2l-1\right)}{\left|D_{c_{k}}\right|}$, for all *l* ∈ *I*_*dc*_*k*__. Both formations move with the same velocity as that of the attackers' center of mass, i.e., ${\dot{\xi }}_{c_{k},l}^{e_{op}}={\dot{\xi }}_{c_{k},l}^{e_{cl}}={\dot{\text{r}}}_{ac_{k}}$.

The defenders $D_{c_{k}}$ first track the desired goal positions $\xi_{c_{k},l}^{e_{op}}$ by using the finite-time convergent, bounded control actions given in Chipade and Panagou (2020b). Once the defender $D_{\beta_{k}\left(1\right)}$ and $D_{\beta_{k}\left(\left|D_{c_{k}}\right|\right)}$ reach within a distance of *b*_*d*_ from $\xi_{c_{k},1}^{e_{op}}$ and $\xi_{c_{k},|D_{c_{k}}|}^{e_{op}}$, i.e., $\left\|{\text{r}}_{d\beta_{k}\left(1\right)}-\xi_{c_{k},1}^{e_{op}}\right\|<b_{d}$ and $\left\|{\text{r}}_{d\beta_{k}\left(\left|D_{c_{k}}\right|\right)}-\xi_{c_{k},|D_{c_{k}}|}^{e_{op}}\right\|<b_{d}$, respectively, the desired goal positions are changed from $\xi_{c_{k},l}^{e_{op}}$ to $\xi_{c_{k},l}^{e_{cl}}$ for all *l* ∈ *I*_*dc*_*k*__. The StringNet is achieved when $\left\|{\text{r}}_{d\beta_{k}\left(l\right)}-\xi_{c_{k},l}^{e_{cl}}\right\|\leq b_{d}$ for all *l* ∈ *I*_*dc*_*k*__ during this phase.

### 3.4. Herding: Moving the Closed-StringNet to Safe Area

Once a group of defenders $D_{c_{k}}$ forms a StringNet around a swarm of attackers $A_{c_{k}}$, they move while tracking a desired rigid closed circular formation 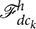 centered at a virtual agent ${\text{r}}_{dc_{k}^{h}}$ as discussed in Chipade and Panagou (2020b). The swarm is herded to the closest safe area *S*_ς(*k*)_, where $\varsigma \left(k\right)=\underset{m\in I_{s}}{\text{arg min}}\left\|{\text{r}}_{dc_{k}^{h}}-{\text{r}}_{sm}\right\|$.

## 4. Multi-Swarm Herding

**Figure 4:**
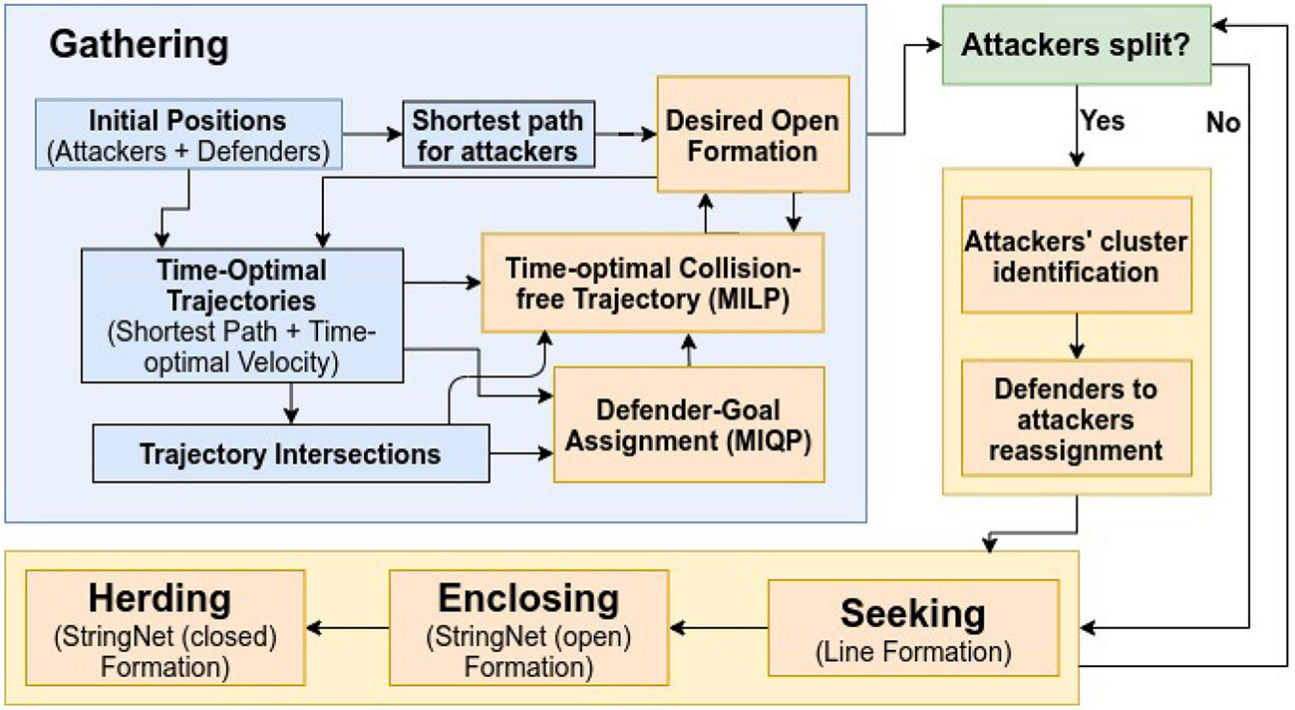
Block diagram for “Multi-Swarm StringNet Herding”.

In this section, we consider that the attackers split into smaller groups as they sense the defenders along their way to the protected area, to maximize the chance of at least some attackers reaching the protected area by circumnavigating the oncoming defenders. To respond to such strategic movements of the attackers, the defenders need to collaborate intelligently. In the approach presented in this paper, as shown in the block diagram in Figure 4, the defenders continuously keep checking whether the attackers have split, i.e., whether the attackers no longer satisfy certain spatial proximity constraints (defined later in the text). After a split event has happened, the defenders first identify the spatial clusters of the attackers. Then, the defenders distribute themselves into smaller connected groups, and these connected groups are assigned to the herd different spatial clusters (swarms) of the attackers to safe areas. Here, by “connected group of defenders" we mean that the defenders have already been connected via string barriers and established an Open-StringNet formation, see for example the defenders at the locations $\left\{\xi_{1}^{g},\xi_{2}^{g},\ldots ,\xi_{5}^{g}\right\}$ and $\left\{\xi_{6}^{g},\xi_{7}^{g},\ldots ,\xi_{10}^{g}\right\}$ as shown in Figure 3. In the next subsections, we discuss how the swarms (clusters) of the attackers are identified and how the defenders are assigned to these identified swarms of the attackers.

### 4.1. Identifying Swarms of the Attackers

In order to identify the spatially distributed clusters (swarms) of the attackers, the defenders utilize the Density Based Spatial Clustering of Applications with Noise (DBSCAN) algorithm (Ester et al., 1996). Given a set of points, DBSCAN algorithm finds clusters of high density points (i.e., points with many nearby neighbors), and marks the points as outliers if they lie alone in low-density regions (whose nearest neighbors are too far away). DBSCAN algorithm can identify clusters of any shape in the data and requires two parameters that define the density of the points in the clusters: (1) ε_*nb*_ (radius of the neighborhood of a point), (2) *m*_*pts*_ (minimum number of points in ε_*nb*_-neighborhood of a point). In general, attackers can split into formations with varied range of densities making the choice of the parameters ε_*nb*_ and *m*_*pts*_ challenging. There are variants of the DBSCAN algorithm, such as OPTICS (Ankerst et al., 1999), which can find clusters of varying density; however, they are more time consuming. Therefore, to keep the computational demands low, we use the DBSCAN algorithm with fixed parameters ε_*nb*_ and *m*_*pts*_, which quickly yields useful clustering information about the attackers satisfying a specified connectivity constraints.

The neighborhood of an attacker is defined using weighted distance between two attackers: $d\left({\text{x}}_{ai},{\text{x}}_{ai^{\prime }}\right)=\sqrt{{\left({\text{x}}_{ai}-{\text{x}}_{ai^{\prime }}\right)}^{T}\text{M}\left({\text{x}}_{ai}-{\text{x}}_{ai^{\prime }}\right)}$, where ${\text{x}}_{ai}={\left[{\text{r}}_{ai}^{T},{\text{v}}_{ai}^{T}\right]}^{T}$ and **M** is a weighing matrix defined as **M** = *diag*([1, 1, φ, φ]), where φ weights relative velocity against relative position. We choose φ < 1 because relative position is more important in a spatial cluster than the velocity alignment at a given time instance. The ε_*nb*_-neighborhood of an attacker $A_{i}$ is then defined as the set of points $\text{x}\in {\mathbb{R}}^{2}\times B_{{\overset{-}{v}}_{a}}$ such that *d*(**x**_*ai*_, **x**) < ε_*nb*_.

The largest circle inscribed in the largest Closed-StringNet formation formed by the 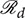
(*N*_*a*_) defenders has radius 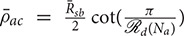. Maximum radius of any cluster with *N*_*a*_ points identified by DBSCAN algorithm with parameters ε_*nb*_ and *m*_*pts*_ is $\frac{\varepsilon_{nb}\left(N_{a}-1\right)}{m_{pts}-1}$. If all of the attackers were to be a single swarm enclosed inside the region with radius ${\overset{-}{\rho }}_{ac}$, then we would require ε_*nb*_ to be greater than $\frac{{\overset{-}{\rho }}_{ac}\left(m_{pts}-1\right)}{N_{a}-1}$ in order to identify them as a single cluster. So we choose $\varepsilon_{nb}=\frac{{\overset{-}{\rho }}_{ac}\left(m_{pts}-1\right)}{N_{a}-1}$, and since we want to identify clusters with as low as 3 agents, we need to choose *m*_*pts*_ = 3. With these parameters for DBSCAN algorithm, we have:

**Lemma 1**. *Let* $A_{c}\left(t\right)=\left\{A_{c_{1}}\left(t\right),A_{c_{2}}\left(t\right),\ldots ,A_{c_{N_{ac}\left(t\right)}}\left(t\right)\right\}$ *be the clusters identified by DBSCAN algorithm with* $\varepsilon_{nb}=\frac{{\overset{-}{\rho }}_{ac}\left(m_{pts}-1\right)}{\left(N_{a}-1\right)}$ *at time *t*, where *N*_*ac*_(*t*) is the total number of clusters at time *t*. If* $|A_{c_{k}}\left(t\right)|>3$ *and *N*_*a*_ = *N*_*d*_, then the radius ρ_*ac*_*k*__(*t*) of the cluster* $A_{c_{k}}\left(t\right)$ *satisfies* 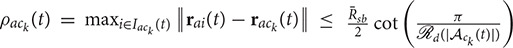, *for all* *k* ∈ *I*_*ac*_(*t*) = {1, 2, …, *N*_*ac*_(*t*)}.

As the number of attackers increases, the computational cost for DBSCAN becomes higher and looses its practical usefulness. Furthermore, the knowledge of the clusters is only required by the defenders when a swarm of attackers does not satisfy the assumed constraint on its connectivity radius so the defenders can be reconfigured and reassigned. So we continuously track the radii of the clusters and run the DBSCAN algorithm only when at some instant *t* = *t*_*se*_ the connectivity constraint is violated by the swarms of attackers $A_{c_{k}}\left(t_{se}\right)$ for some *k* ∈ *I*_*ac*_(*t*_*se*_) i.e., when the radius ρ_*ac*_*k*__(*t*_*se*_) of the swarm of attackers $A_{c_{k}}\left(t_{se}\right)$ exceeds the value 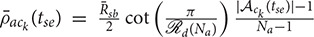. The connectivity constraint violation is termed as split in this paper. The split event is defined as:

**Definition 4** (Split event). *An instant* *t*_*se*_ *when for any swarm* $A_{c_{k}}\left(t_{se}\right)$, *k* ∈ *I*_*ac*_(*t*_*se*_), *the radius of the swarm of attackers* $A_{c_{k}}\left(t_{se}\right)$ *defined as* $\rho_{ac_{k}}\left(t_{se}\right)=\max_{i\in I_{ac_{k}}\left(t_{se}\right)}\left\|{\text{r}}_{ai}\left(t_{se}\right)-{\text{r}}_{ac_{k}}\left(t_{se}\right)\right\|$ *exceeds the value* ${\overset{-}{\rho }}_{ac_{k}}\left(t_{se}\right)$.

We also make the following assumption regarding the splitting behavior of the attackers.

**Assumption 2**. *Once a swarm of attackers splits, its member attackers never rejoin each other, i.e., for all* *i* ∈ *I*_*a*_, *if* ∃ *t* > *0 such that* $A_{i}\notin A_{c_{k}}\left(t\right)$ *for any* *k* ∈ *I*_*ac*_(*t*) *then* $A_{i}\notin A_{c_{k}}\left(t^{\prime }\right)$ *for all* *t* ≤ *t*′.

### 4.2. Defender Assignment to the Swarms of Attackers

The initially single-one swarm of attackers splits into smaller swarms that are being identified by the defenders. After then, the defenders must distribute themselves into smaller groups, and assign these groups to the attackers' swarms (clusters), so that subsequently they enclose the attackers' clusters and herd them to the closest safe area. Let $A_{c}\left(t_{se}\right)=\left\{A_{c_{1}}\left(t_{se}\right),A_{c_{2}}\left(t_{se}\right),\ldots ,A_{c_{N_{ac}}}\left(t_{se}\right)\right\}$ be a set of swarms of the attackers after a split event has happened at time *t*_*se*_.

We assume that none of the swarms in $A_{c}\left(t\right)$ is a singular one, i.e., a swarm with less than three agents, $|A_{c_{k}}\left(t\right)|>2$ for all *k* ∈ *I*_*ac*_(*t*), *t* ≥ 0. We formally define the defender to attackers' swarm assignment as:

**Definition 5** (Defender-to-AttackSwarm Assignment). *A set* β(*t*) = {β_1_(*t*, ·), β_2_(*t*, ·), …β_*N*_*ac*__(*t*, ·)} *of mappings* β_*k*_(*t*, ·):{1, 2, …, 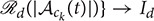, *where* β_*k*_(*t*, ·) *gives the indices of the defenders assigned to the swarm* $A_{c_{k}}\left(t\right)$ *at time* *t* *for all* *k* ∈ *I*_*ac*_(*t*).

We want to find an assignment that minimizes the sum of distances of the defenders from the centers of the assigned attackers' swarms. This ensures that the collective effort needed by all the defenders is minimized when enclosing the swarms of the attackers. For successful enclosing of the newly formed attacking swarms, it is required that all the defenders that are assigned to an attackers' cluster are neighbors of each other, are already connected to each other via string barriers, and the underlying graph is an Open-StringNet.

Let $D_{c}\left(t_{se}^{-}\right)=\left\{D_{c_{1}}\left(t_{se}^{-}\right),D_{c_{2}}\left(t_{se}^{-}\right),\ldots ,D_{c_{N_{dc}\left(t_{se}^{-}\right)}}\left(t_{se}^{-}\right)\right\}$ be a set of swarms of the defenders, where $t_{se}^{-}$ denotes the instant immediately before *t* = *t*_*se*_. Each of these swarms is already connected via a StringNet and was assigned to herd some cluster of attackers an instant before a split event happened at time *t*_*se*_. Now that a split event has happened and new smaller clusters have been formed by the attackers, we seek to reassign the defenders $D_{c}\left(t_{se}^{-}\right)$ to herd the newly formed clusters of the attackers.

This assignment problem is closely related to generalized assignment problem (GAP) (Öncan, 2007), in which *n* objects are to be filled in *m* knapsacks (*n* ≥ *m*). This problem is modeled as a GAP with additional constraints on the objects (defenders) that are assigned to a given knapsack (attackers' swarm). The additional constraint on the defenders is to ensure their connectivity to each other within a newly formed swarm of defenders. So, we call this constrained assignment problem as connectivity constrained generalized assignment problem (C2GAP), and provide a mixed integer quadratically constrained program (MIQCP) to find the optimal assignment centrally as:


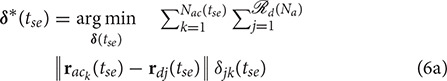


(6b) $$\begin{array}{ll} \text{Subject to } & \sum_{k\in I_{ac}\left(t_{se}\right)}\delta_{jk}\left(t_{se}\right)=1,\;\forall j\in I_{dc_{0}}; \end{array}$$

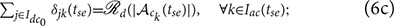



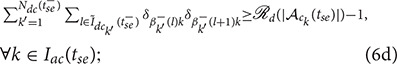



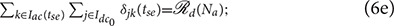


(6f) $$\begin{array}{l} \delta_{jk}\left(t_{se}\right)\in \left\{0,1\right\},\;\forall j\in I_{dc_{0}},k\in I_{ac}\left(t_{se}\right); \end{array}$$

where *I*_*dc*_0__ = {1, 2, …, 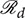
(*N*_*a*_)}; ${\tilde{I}}_{dc_{k^{\prime }}}\left(t_{se}^{-}\right)=\left\{1,2,\ldots ,|D_{c_{k^{\prime }}}\left(t_{se}^{-}\right)|-1\right\}$, where $D_{c_{k^{\prime }}}\left(t_{se}^{-}\right)$ is the *k*^′*th*^ swarm before the reassignment; 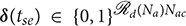 is the binary decision vector defined as **δ**(*t*_*se*_) = {δ_*jk*_(*t*_*se*_)|*j* ∈ *I*_*d*_*c*__0__, *k* ∈ *I*_*ac*_(*t*_*se*_)}, where δ_*jk*_(*t*_*se*_) is a decision variable which is equal to 1 when the defender $D_{j}$ is assigned to the swarm $A_{c_{k}}\left(t_{se}\right)$ and 0 otherwise; and $\beta_{k^{\prime }}^{-}\left(\cdot \right)=\beta_{k^{\prime }}\left(t_{se}^{-},\cdot \right)$ is the assignment of the defenders to a cluster $A_{c_{k^{\prime }}}\left(t_{se}^{-}\right)$ prior to the split event. The constraints (6b) ensure that each defender is assigned to exactly one swarm of the attackers. The capacity constraints (6c) ensure that for all *k* ∈ *I*_*ac*_(*t*_*se*_) swarm $A_{c_{k}}\left(t_{se}\right)$ has exactly 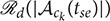 defenders assigned to it. The quadratic constraints (6d) ensure that all the defenders assigned to swarm $A_{c_{k}}\left(t_{se}\right)$ are connected together with an underlying Open-StringNet for all *k* ∈ *I*_*ac*_(*t*_*se*_). Consider any sub-group of 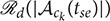 defenders out of the defenders that are already connected via Open-StringNet at the time of assignment. The intuition behind the constraints (6d) is that, for these 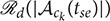 defenders, to be connected to each other via an Open-StringNet, each defender needs to be connected to its immediate neighbors and the total number of such connections should be equal 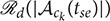 − 1. If the total number of connections is less than 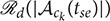 − 1 then it is evident that there are at least two disconnected components in the given subgroup of defenders and they do not form a single Open-StringNet, which is not desired in our case. For example, in the scenario shown in Figure 3, if we were to assign 5 defenders to a cluster of attackers then the only choices under this quadratic constraint would be to choose the defenders at $\xi_{l}^{g}$ for *l* ∈ {1, 2, …, 5} or the defenders at $\xi_{l}^{g}$ for *l* ∈ {6, 7, …, 10} and not the defenders at $\xi_{l}^{g}$ for *l* ∈ {1, 2, 7, 8, 9} or any such combination of defenders that violates the Open-StringNet connectivity. The constraint (6e) ensures that all the 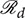
(*N*_*a*_) defenders are assigned to the attackers' swarms.

The aforementioned MIQCP can be solved using a MIP solver Gurobi (Gurobi Optimization, 2018). After solving (6), one can find the mapping β_*k*_(*t*, ·), for all *k* ∈ *I*_*ac*_(*t*), as follows:

(7) $$\begin{array}{l} \beta_{k}\left(t,l\right)=\beta_{k^{*}}^{-}\left(l_{0}+l\right),\text{ for }t\in \left[t_{se}+t_{comp},\min\left(t_{se}^{next},\infty \right)\right], \end{array}$$

where $k^{*}={\text{arg max}}_{k^{\prime }}\overset{\left|D_{c_{k^{\prime }}}\left(t_{se}^{-}\right)\right|}{\underset{l^{\prime }}{\sum }}\delta_{\beta_{k^{\prime }}^{-}\left(l^{\prime }\right)k}\left(t_{se}\right)$ and *l*_0_ is the smallest integer for which $\delta_{\beta_{k^{*}}^{-}\left(l_{0}+1\right)k}\left(t_{se}\right)=1$; *t*_*comp*_ is the computation time to solve (6); and $t_{se}^{next}$ is an unknown future time at which a split happens. In other words, the assignment obtained using the states at *t*_*se*_ continues to be a valid assignment until the next split event happens at some unknown time $t_{se}^{next}$ in the future. As shown in an instance of the defender-swarm assignment in Figure 3, the defenders at $\xi_{l}^{g}$ for *l* ∈ {1, 2, …, 5} are assigned to swarm $A_{c_{2}}\left(t_{se}\right)$ and those at $\xi_{l}^{g}$ for *l* ∈ {6, 7, …, 10} are assigned to swarm $A_{c_{1}}\left(t_{se}\right)$.

### 4.3. Decentralized Algorithm for Defender to AttackSwarm Assignment

The MIQCP in (6) is solved centrally, i.e., a single agent has access to the information pertaining to all the agents and the MIQCP is solved by a single agent or computer and the assignment result is communicated to other agents. To make the proposed assignment approach robust toward failure, we provide a decentralized algorithm to solve the assignment problem.

When a swarm of attackers $A_{c_{k}}$ splits into smaller swarms at *t* = *t*_*se*_. The newly identified swarms of the attackers by the DBSCAN algorithm are assigned new indices. Namely, one of the swarm is assigned the index *k*, i.e., the index of the parent swarm $A_{c_{k}}$ and the rest swarms are assigned integers greater than *N*_*ac*_(*t*_*se*_) as their indices. Let $C^{\left(k\right)}\left(t_{se}\right)$ denote the indices of the clusters of the attackers that are newly formed out of the parent cluster $A_{c_{k}}\left(t_{se}^{-}\right)$, when the cluster $A_{c_{k}}$ splits at *t* = *t*_*se*_, as identified by the DBSCAN algorithm. Then, we can assign the defenders in $D_{c_{k}}\left(t_{se}^{-}\right)$ only to the clusters $A_{c_{k}^{\prime }}\left(t_{se}\right)$, for all $k^{\prime }\in C^{\left(k\right)}\left(t_{se}\right)$, and not consider other clusters of attackers, that did not split, or the other defenders during the reassignment process. We do so by solving the following modified MIQCP, which is solved only for the defenders in $D_{c_{k}}\left(t_{se}^{-}\right)$ and the attackers in the cluster $A_{c_{k}}\left(t_{se}^{-}\right)$.

(8a) $$\begin{array}{l} \delta^{\left(k\right)*}\left(t_{se}\right)=\underset{\delta^{\left(k\right)}\left(t_{se}\right)}{\text{arg min}}\;\underset{k^{\prime }\in C^{\left(k\right)}\left(t_{se}\right)}{\sum }\\ \;\underset{j\in D_{c_{k}}\left(t_{se}^{-}\right)}{\sum }{\|\text{r}}_{ac_{k^{\prime }}}\left(t_{se}\right)-{\text{r}}_{dj}\left(t_{se})\right\|\delta_{jk^{\prime }}\left(t_{se}\right) \end{array}$$

(8b) $$\begin{array}{ll} \text{Subject to } & \sum_{k^{\prime }\in C^{\left(k\right)}\left(t_{se}\right)}\delta_{jk^{\prime }}\left(t_{se}\right)=1,\;\forall j\in D_{c_{k}}\left(t_{se}^{-}\right); \end{array}$$

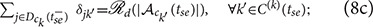



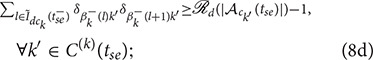


(8e) $$\begin{array}{l} \sum_{j\in D_{c_{k}}\left(t_{se}^{-}\right)}\sum_{k^{\prime }\in C^{\left(k\right)}\left(t_{se}\right)}\delta_{jk^{\prime }}=|D_{c_{k}}\left(t_{se}^{-}\right)|; \end{array}$$

(8f) $$\begin{array}{l} \delta_{jk}\left(t_{se}\right)\in \left\{0,1\right\},\;\forall j\in D_{c_{k}}\left(t_{se}^{-}\right),k\in C^{\left(k\right)}\left(t_{se}\right); \end{array}$$

where $\delta^{\left(k\right)}\left(t_{se}\right)\in {\left\{0,1\right\}}^{\left|D_{c_{k}}\left(t_{se}^{-}\right)\|C^{\left(k\right)}\left(t_{se}\right)\right|}$ defined as $\delta^{\left(k\right)}\left(t_{se}\right)=\left[\delta_{jk^{\prime }}\left(t_{se}\right)|j\in D_{c_{k}}\left(t_{se}^{-}\right),k^{\prime }\in C^{\left(k\right)}\left(t_{se}\right)\right]$, $D_{c_{k}}\left(t_{se}^{-}\right)=\left\{\beta_{k}\left(t_{se}^{-},j\right)|j\in \left\{1,2,\ldots ,|D_{c_{k}}\left(t_{se}^{-}\right)|\right\}\right\}$ is the set of indices of the defenders in $D_{c_{k}}$ prior to the reassignment. As one can see, the dimension of the decision vector $\delta^{\left(k\right)}\left(t_{se}\right)$ is going to be smaller than that of the decision vector in (6) and hence (8) can be solved relatively quicker than (6).

Although the clustering information is acquired and tracked centrally, the problem in (8) is solved and the assignment solution is communicated to other teammates by the lead defender in $D_{c_{k}}\left(t_{se}^{-}\right)$, where the lead defender is identified to be the one in the middle of the Open-StringNet formation, i.e., the defender $D_{\beta_{k}(t_{se}^{-},l_{i})}$ where $l_{i}=\left\lfloor \frac{\left|D_{c_{k}}\left(t_{se}^{-}\right)\right|}{2}\right\rfloor$, for all *k* for which the $A_{c_{k}}$ have split.

This helps the defenders find the Defender-to-AttackSwarm assignment quickly, and without having to consider all the agents in the assignment formulation, i.e., in a decentralized way.

If only one swarm of the attackers splits at a given time instant *t*_*se*_ then indeed the optimal cost obtained in (6) and (8) are same. For other cases, the cost obtained by the decentralized algorithm would be suboptimal. A detailed analysis of performance of the centralized and the decentralized algorithms will be studied in our future work.

### 4.4. Heuristic to Find Defender-To-AttackSwarm Assignment

**Figure 5:**
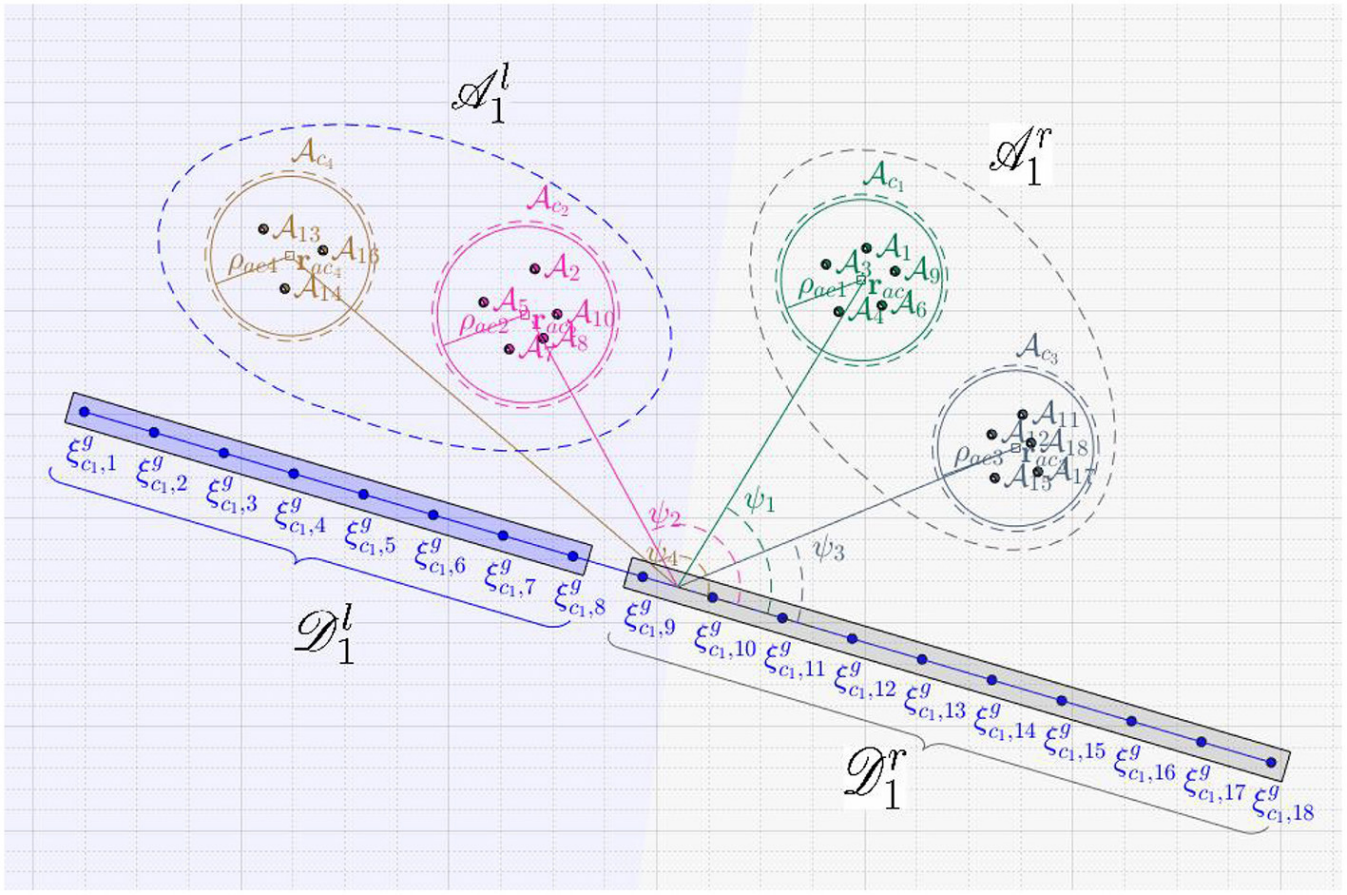
Splitting for the hierarchical algorithm.

Finding the optimal defender-swarm assignment by solving the MIQCPs discussed above may not be real-time implementable for a large number of agents (>100). In this section, we develop a computationally-efficient heuristic, called hierarchical approach, to find defender-swarm assignment. A large dimensional assignment problem is split into smaller, lower-dimensional assignment problems that can be solved optimally and quickly using the MIQCP formulation discussed earlier. Algorithm 1 provides the steps to reduce the problem of size *N*_*ac*_ to smaller problems of size smaller than or equal *N*_*ac*_(≤ *N*_*ac*_). In Algorithm 1, 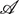 is a data structure, at the time of assignment *t* = *t*_*se*_, with fields that store the information of: (i) centers of the attackers' swarms ${\text{R}}_{a}\left(t_{se}\right)=\left[{\text{r}}_{{ac}_{k^{\prime }}}\left(t_{se}\right)|k^{\prime }\in C^{\left(k\right)}\left(t_{se}\right)\right]$, (ii) numbers of the attackers in each newly formed swarm ${\text{n}}_{a}\left(t_{se}\right)=\left[|A_{c_{k^{\prime }}}\left(t_{se}\right)\|k^{\prime }\in C^{\left(k\right)}\left(t_{se}\right)\right]$, (iii) total number of attackers $N_{a}^{-}=N_{ac_{k}}\left(t_{se}^{-}\right)$. Similarly, 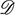 is a data structure that stores the information of: (i) defenders' positions ${\text{R}}_{d}\left(t_{se}^{-}\right)=\left\{{\text{r}}_{dj}\left(t_{se}^{-}\right)|j\in D_{c_{k}}\left(t_{se}^{-}\right)\right\}$, and (ii) the goal assignment $\beta^{-}=\beta_{k}\left(t_{se}^{-},\cdot \right)$. splitApproxEqual function splits the attackers into two groups 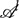 and 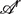 of roughly equal number of attackers, and the defenders into two groups 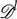 and 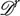. The split is performed based on the angles $\psi_{k^{\prime }}$ made by relative vectors ${\text{r}}_{ac_{k^{\prime }}}\left(t_{se}\right)-{\text{r}}_{dc}\left(t_{se}^{-}\right)$, for all $k^{\prime }\in C^{\left(k\right)}\left(t_{se}\right)$, with the vector ${\text{r}}_{d\beta^{-}\left(\left|D_{c_{k}}\left(t_{se}^{-}\right)\right|\right)}\left(t_{se}\right)-{\text{r}}_{dc}\left(t_{se}^{-}\right)$ where ${\text{r}}_{dc}\left(t_{se}^{-}\right)$ is the center of ${\text{R}}_{d}\left(t_{se}^{-}\right)$ as shown in Figure 5.

We first arrange these angles $\psi_{k^{\prime }}$ in descending order. The first few clusters in the arranged list with roughly half the total number of attackers become the left group 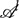 and the rest become the right group 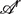. Similarly, the left group 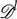 is formed by the first 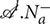 defenders as per the assignment β^−^ and the rest defenders form the right group 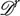. For example, as shown in Figure 5, after the very first split event, the clusters $A_{c_{1}},A_{c_{3}}$ become part of 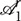 and $A_{c_{2}},A_{c_{4}}$ are a part of 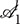. Similarly, the defenders at $\xi_{c_{1},j}^{g}$ for *j* ∈ {1, 2, …, 8} become part of 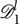 while the defenders at $\xi_{c_{1},j}^{g}$ for *j* ∈ {9, 10, …, 18} become part of 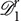. We assign the defenders in 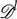 only to the swarms in 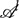 and those in 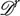 only to the swarms in 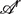. By doing so we may or may not obtain an assignment that minimizes the cost in (6a) but we reduce the computation time significantly and obtain a reasonably good assignment quickly. As in Algorithm 1, the process of splitting is done recursively until the number of attackers' swarms is smaller than a pre-specified number *N*_*ac*_. The function assignMIQCP finds the defender-swarm assignment by solving (6).

**Algorithm 1:**
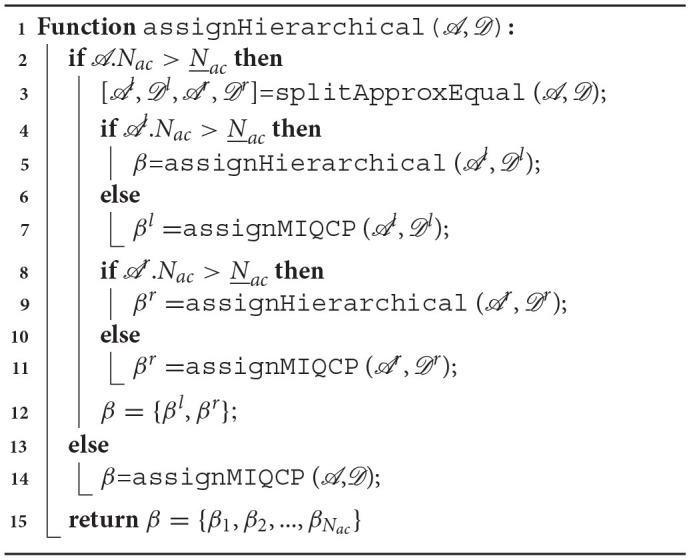
Defender-Swarm Assignment

**Figure 6:**
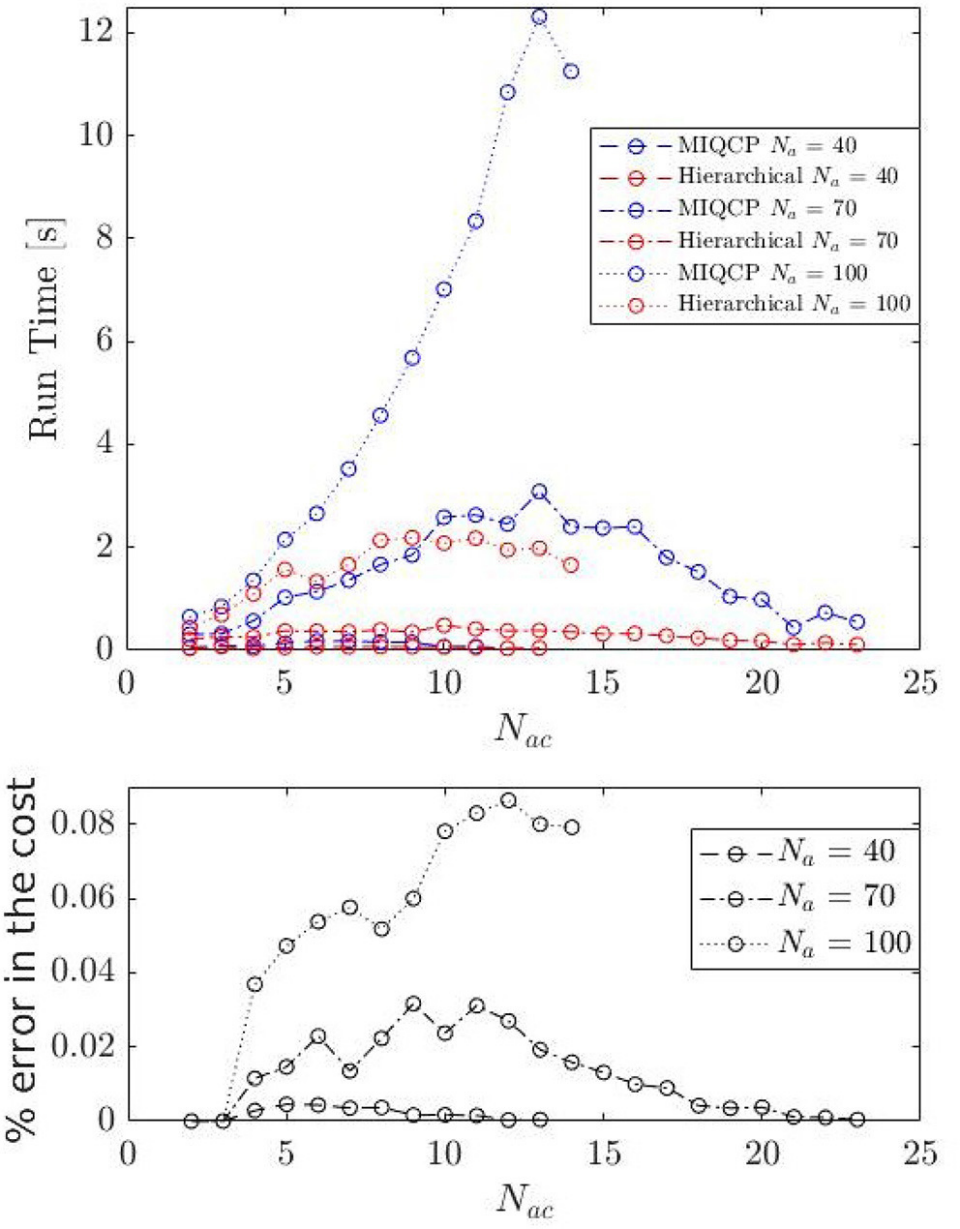
Run-time for assignment algorithms.

In Figure 6, we show the average computation time for a number of cluster configurations and random initial conditions. The computation cost first increases with the number of clusters, reaches a maximum point, and then decreases. This is because the computation cost is proportional to the number of choices available, i.e., number of ways *N*_*ac*_ groups of given sizes can be formed out of *N* players (CNNac). More importantly, as shown in Figure 6, the average computation time for the hierarchical approach (heuristic) to assignment is significantly smaller than that of the MIQCP formulation. We also compare the cost of MIQCP and the heuristic. Figure 6 shows the percentage error between the cost from heuristic and the optimal cost from MIQCP, defined as $\text{\% error}=\frac{100|cost_{MIQCP}-cost_{Heuristic}|}{cost_{MIQCP}}$, where *cost*_*MIQCP*_ and *cost*_*Heuristic*_ are the costs obtained by the MIQCP and the Heuristic, respectively. The cost of the hierarchical algorithm is very close to the optimal cost (MIQCP), see Figure 6. In summary, the hierarchical heuristic exploits the geometry to provide a reasonable assignment solution within a fraction of time that the optimal MIQCP formulation could have taken to find.

## 5. Dominance Region for the Defenders

In this and the following section, we provide conditions under which the defenders can successfully herd the attackers. The defenders succeed in herding the attackers if they manage to achieve the open-StringNet with line formation 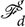 centered at a gathering center on the expected path of the attackers, well before the attackers reach the gathering center. If the successful gathering is possible by the defenders, then they could proceed to use the proposed StringNet herding approach to herd the attackers to safe areas, otherwise, the defenders would have to use some other approach to defense, such as direct physical capturing of the attackers.

For given initial conditions of all the agents, the defenders require to solve the problem of finding the best gathering center ${\text{r}}_{dc^{g}}$ and the corresponding defender-goal assignment β_*o*_ using the iterative MIQP formulation discussed in Algorithm 1 in Chipade and Panagou (2020b). One needs to check if this problem is feasible for given initial conditions to conclude whether defenders can succeed in gathering. This formulation, however, becomes computationally demanding as the number of agents becomes larger. In order to quickly decide if the successful gathering is feasible or not, we provide the following approximate problem formulation that can be solved relatively quickly.

Let *T*_*a*_(**r**_*a*_, **r**, ρ_*a*_) be the minimum time required by an attacker at **r**_*a*_ to reach within ρ_*a*_ distance from the point **r** ∈ ℝ^2^. Let **R**_*d*_ = [**r**_*d*1_, **r**_*d*2_, …, **r**_*dN*_*d*__] denote the positions of the defenders $D_{j}$ for all *j* ∈ *I*_*d*_. Let 
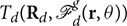
 be the maximum time required by all the defenders to achieve the gathering formation 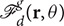 centered at **r** with orientation vector making an angle θ with x-axis.

**Figure 7:**
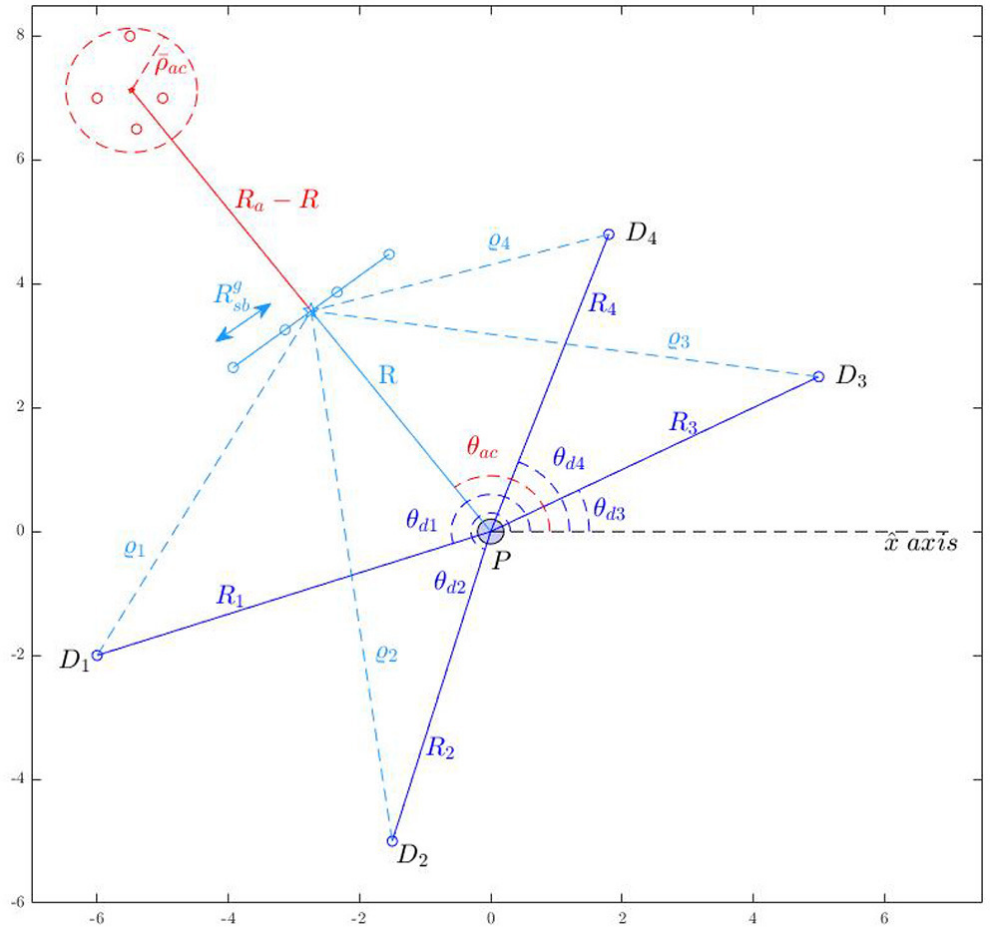
Abstraction for estimate of dominance region.

Consider *N*_*d*_ defenders and *N*_*a*_ attackers located at given positions as shown in Figure 7. Consider the protected area located at the origin (${\text{r}}_{p}={\left[0,0\right]}^{T}$). Let the largest radius of the attackers' formation be ${\overset{-}{\rho }}_{ac}$. Let the position vector of the center of mass of the attackers make an angle θ_*ac*_ with x-axis. Let the center of the desired formation be located at a distance *R* from the protected area along the direction θ_*ac*_. The distance of the defender $D_{j}$ from the center of the desired formation is:

(9) $$\begin{array}{l} \varrho_{j}=\sqrt{R^{2}+R_{j}^{2}-2RR_{j}\cos\left(\left|\theta_{dj}-\theta_{ac}\right|\right)}, \end{array}$$

for all *j* ∈ *I*_*d*_. The maximum value among ϱ_*j*_, for all *j* ∈ *I*_*d*_, can be bounded as: $\overset{-}{\varrho }=\underset{j\in I_{d}}{\max}\varrho_{j}\leq {\tilde{\varrho }}_{\delta }={\left(\underset{j\in I_{d}}{\sum }\varrho_{j}^{\delta }\right)}^{\frac{1}{\delta }}$ (Stipanović et al., 2012). For which we also have $\underset{\delta \to \infty }{\lim}{\tilde{\varrho }}_{\delta }=\bar{\varrho }$.

The maximum distance any defender would have to travel in the best defender-goal assignment can be upper bounded by ${\overset{-}{\varrho }}_{d}={\tilde{\varrho }}_{\delta }+0.5\left(N_{d}-1\right)R_{sb}^{g}$. The maximum time for any defender to reach the gathering location assigned to it as per the best defender-goal assignment under time-optimal control (Chipade and Panagou, 2020a) can be bounded from above by:

(10) $$\begin{array}{l} \begin{array}{c} {\overset{-}{T}}_{d}=\tau \left({\overset{-}{\varrho }}_{d},0,0\right)=\frac{1}{\lambda_{0}}\left(\tanh^{-1}\left(\frac{v_{sw}}{{\overset{-}{v}}_{d}}\right)+\tan^{-1}\left(\frac{v_{sw}}{{\overset{-}{v}}_{d}}\right)\right) \end{array} \end{array}$$

where $\lambda_{0}=\sqrt{{\bar{u}}_{d}C_{D}}$, $v_{sw}=\sqrt{\frac{\left(\lambda -1\right){\bar{u}}_{d}}{\left(\lambda +1\right)C_{D}}}$, $\lambda =e^{2C_{D}{\overset{-}{\varrho }}_{d}}$. Similarly, the minimum time that the attackers require to reach the gathering location is when the attackers move toward the protected with the maximum possible speed. Then, the difference between the time needed for the attackers to reach the gathering location and the time required by the defenders to reach there can be bounded from below by:

(11) $$\begin{array}{l} \Delta T=\frac{R_{ac}-{\overset{-}{\rho }}_{ac}-R}{{\overset{-}{v}}_{a}}-{\overset{-}{T}}_{d}\left(R\right) \end{array}$$

where *R*_*ac*_ is the distance of the center of mass of the attackers from the center of the protected area and ${\overset{-}{\rho }}_{ac}$ is the maximum radius of the attackers' formation under consideration. Defenders want $\Delta T\geq \Delta T_{d}^{g}$ to be able to gather well before the attackers reach the gathering center. Here $\Delta T_{d}^{g}$ is a user-defined time to account for the size of the attackers' swarm and the time required by the defenders to get connected by string barriers once arrived at the desired gathering formation. Given initial states of the attackers, one can find Δ*T* using (11) to assess, at least conservatively, whether the defenders can gather in the attackers' path before the attackers, without solving the actual, computationally heavy iterative MIQP formulation (Chipade and Panagou, 2020b).

Furthermore, using the above approximate analysis, for given initial conditions of the defenders, we characterize sufficient conditions on the initial positions of the attackers for which the defenders successfully gather on the shortest path of the attackers to the protected area, before the attackers can reach there. We call this set of initial conditions of the attackers as the dominance region for the given initial positions of the defenders. The dominance region is formally defined as:

**Definition 6** (Defenders' Dominance Region). $Dom\left({\text{R}}_{d},{\overset{-}{\rho }}_{ac},\Delta T_{d}^{g}\right)=\{\text{r}\in {\mathbb{R}}^{2}|\exists \upsilon \in \left(\frac{\rho_{p}}{\left\|\text{r}\right\|},1-\frac{{\overset{-}{\rho }}_{ac}}{\left\|\text{r}\right\|}\right)$ *such that* 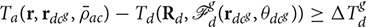 $\text{where }{\text{r}}_{dc^{g}}=\upsilon \text{r}\}$.

We use (11) to find an estimate *Dom*_*est*_ of the dominance region *Dom* that is completely contained inside *Dom*. We are interested in the limiting condition when $\Delta T=\Delta T_{d}^{g}$, that corresponds to the boundary of *Dom*_*est*_, for which we have:

(12) $$\begin{array}{l} R_{ac}=f\left(R\right)={\overset{-}{\rho }}_{ac}+R+{\overset{-}{v}}_{a}\left({\overset{-}{T}}_{d}\left(R\right)+\Delta T_{d}^{g}\right). \end{array}$$

We want to find the smallest value *R*_*ac*_(> ρ_*p*_) of *R*_*ac*_ for which $\Delta T=\Delta T_{d}^{g}$, i.e.,

(13) $$\begin{array}{l} {\underline{R}}_{ac}=\min_{R>\rho_{p}}f\left(R\right). \end{array}$$

**Lemma 2**. *Given that no two defenders are co-located, i.e.,* $\left\|{\text{r}}_{dj}-{\text{r}}_{dj^{\prime }}\right\|>0$ *for all* $j\neq j^{\prime }\in I_{d}$, *f*(*R*) *as given in Equation (12) is a locally convex function of* *R*.

*Proof*: The proof is provided in the Appendix.     □

**Figure 8:**
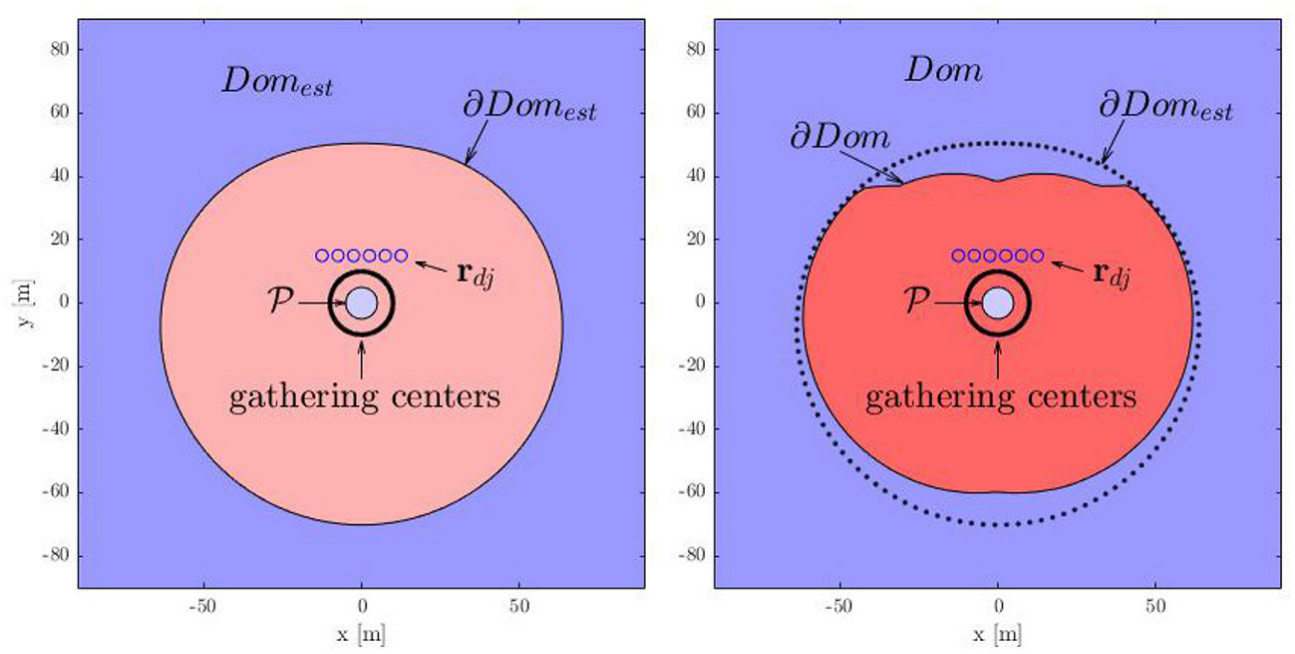
Dominance regions of the players.

One can find *R*_*ac*_ by solving the convex optimization (13) with *R* = R^*^, the minimizer of ${\tilde{\varrho }}_{\delta }\left(R\right)$, as an initial guess to a gradient descent algorithm with sufficiently small step size. For different directions from which the attackers can approach the protected area, we solve the convex optimization (13) to find the corresponding point on the boundary *Dom*_*est*_. Figure 8 shows the boundaries ∂*Dom*_*est*_ and ∂*Dom* of the estimate *Dom*_*est*_ and the dominance region *Dom*, respectively. Here ∂*Dom* is obtained by numerically evaluating the iterative MIQP for each direction. The regions outside of the closed boundaries ∂*Dom*_*est*_ and ∂*Dom* are, respectively, *Dom*_*est*_ and *Dom*, computed for the case where the defenders are at given locations (blue circles). On the other hand, the set inside the boundaries ∂*Dom*_*est*_ and ∂*Dom* are the complement sets $Dom_{est}^{c}={\mathbb{R}}^{2}\backslash Dom_{est}$ and *Dom*^*c*^ = ℝ^2^\*Dom*, respectively. The set *Dom*^*c*^ is essentially the dominance region of the attackers, i.e., the attackers can reach the protected area before the defenders can gather on their path if the attackers start inside *Dom*^*c*^. Note that the estimate *Dom*_*est*_ is completely contained in the dominance region *Dom*. The region *Dom* is larger on the side where the density of the defenders is larger. This is intuitive because many defenders have to travel less when the attackers approach from this side and hence allow defenders to gather on the expected path of the attackers in time even if the attackers start more closer to the protected area on this side. We have the following result based on the above analysis.

**Theorem 3**. *Consider a group of defenders* $D_{c}=\left\{D_{1},D_{2},\ldots D_{N_{dc}}\right\}$ *starting at given locations* **R**_*dc*_ = [**r**_*d*1_, **r**_*d*2_, …, **r**_*dN*_*d*__] *and a swarm of Attackers* $A_{c}$ *with footprint of maximum radius* ${\overset{-}{\rho }}_{ac}$. *If the attackers start inside* $Dom_{est}\left({\text{R}}_{dc},{\overset{-}{\rho }}_{ac},\Delta T_{d}^{g}\right)$, *then the defenders in* $D_{c}$ *are guaranteed to achieve a formation* 
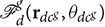
 *on the shortest path from the center of mass of the attackers in* $A_{c}$ *to the protected area* P *before the attackers could reach there*.

*Proof*: By construction, $Dom_{est}\left({\text{R}}_{dc},{\overset{-}{\rho }}_{ac},\Delta T_{d}^{g}\right)\subseteq Dom\left({\text{R}}_{dc},{\overset{-}{\rho }}_{ac},\Delta T_{d}^{g}\right)$. The proof follows from the definition of the dominance region $Dom\left({\text{R}}_{dc},{\overset{-}{\rho }}_{ac},\Delta T_{d}^{g}\right)$.     □

In other words, Theorem 3 states that for the attackers starting in $Dom_{est}\left({\text{R}}_{d},{\overset{-}{\rho }}_{ac},\Delta T_{d}^{g}\right)$, the defenders are guaranteed to gather in their shortest path to the protected area in time. However, if the attackers do not start in $Dom_{est}\left({\text{R}}_{d},{\overset{-}{\rho }}_{ac},\Delta T_{d}^{g}\right)$ nothing can be concretely said about the gathering of the defenders based on the above approximate analysis.

## 6. Results

Based on the DBSCAN clustering based assignment algorithm discussed earlier and the conditions on the initial states of the agents for successful gathering, we have the following result on the successful herding of the attackers.

**Theorem 4**. *Consider a group of defenders* 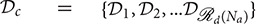 *starting at given locations* **R**_*dc*_ = [**r**_*d*1_, **r**_*d*2_, …, **r**_*d*
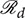
(*N*_*a*_)_]. *Let attackers' initial positions be such that* $\rho_{ac}\left(0\right)=\max_{i\in I_{a}}\|{\text{r}}_{ai}\left(0\right)-{\text{r}}_{ac}\left(0\right)\|\leq {\overset{-}{\rho }}_{ac}$, *where* ${\text{r}}_{ac}\left(0\right)=\underset{i\in I_{a}}{\sum }\frac{{\text{r}}_{ai}\left(0\right)}{N_{a}}$, *and that they belong to* $Dom_{est}\left({\text{R}}_{dc},{\overset{-}{\rho }}_{ac}\right)$. *Furthermore, suppose that for all times* *t* > 0, *each attacker belongs to one of the clusters* $\left\{A_{c_{1}},A_{c_{2}},\ldots ,A_{c_{N_{ac}}}\right\}$, *for some* *N*_*ac*_, *that are identified by DBSCAN algorithm with* $\varepsilon_{nb}=\frac{{\overset{-}{\rho }}_{ac}\left(m_{pts}-1\right)}{N_{a}-1}$ *and* *m*_*pts*_ = 3 *whenever a swarm splits into smaller swarms. Then*,

*(i) the defenders in* $D_{c}$ *are guaranteed to enclose every attacker inside a StringNet; and*
*(ii) if a swarm of attackers has not reached the protected area* P *by the time it is enclosed within a StringNet then the swarm is guaranteed to be herded to one of the safe areas*.

*Proof*: *(i)* The attackers start inside $Dom_{est}\left({\text{R}}_{dc},{\overset{-}{\rho }}_{ac}\right)\subset Dom\left({\text{R}}_{dc},{\overset{-}{\rho }}_{ac}\right)$, so as per Theorem 3 the defenders are able to gather and get connected by string barriers on the shortest path of the attackers to the protected area. Since each attacker belongs to one of the swarms of attackers $\left\{A_{c_{1}},A_{c_{2}},\ldots ,A_{c_{N_{ac}}}\right\}$ that is identified by DBSCAN algorithm, we have from the Lemma 1 that each cluster satisfies 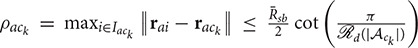, as long as $|A_{c_{k}}|>3$. This implies that a group of 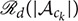 defenders $D_{c_{k}}$ connected via StringNet are capable of enclosing the attackers in $A_{c_{k}}$ for all *k* ∈ *I*_*ac*_. Under the control laws as described in section 3 (and in Chipade and Panagou (2020b)), it is proved in Theorem 5 and 6 in Chipade and Panagou (2020b) that the defenders in $D_{c_{k}}$ form a closed-StringNet around the attackers in $A_{c_{k}}$, i.e., the defenders enclose the attackers, for all *k* ∈ *I*_*ac*_.
*(ii)* Once the attackers in a cluster $A_{c_{k}}$ are enclosed by the defenders in $D_{c_{k}}$ and it is true that the attackers have not reached the protected area, then the defenders in $D_{c_{k}}$, under the control actions described in section 3.4 (and in Chipade and Panagou 2020b), herd the attackers in $A_{c_{k}}$ to one of the safe areas as proved in Theorem 7 in Chipade and Panagou (2020b), for all *k* ∈ *I*_*ac*_.     □

**Remark 1**. *We do provide strong guarantees on the completion of the gathering phase. We also proved in Chipade and Panagou (2020b) that the attackers' clusters will be enclosed within some finite time under the state-feedback control laws as discussed in Chipade and Panagou (2020b). However, finding upper bounds on this finite time with many agents and formation interacting with each other is not a trivial task and hence providing strong conditions under which seeking and enclosing phases are also completed well before attackers reach the protected areas for each cluster of attackers is left open for future research*.

## 7. Simulations

**Figure 9:**
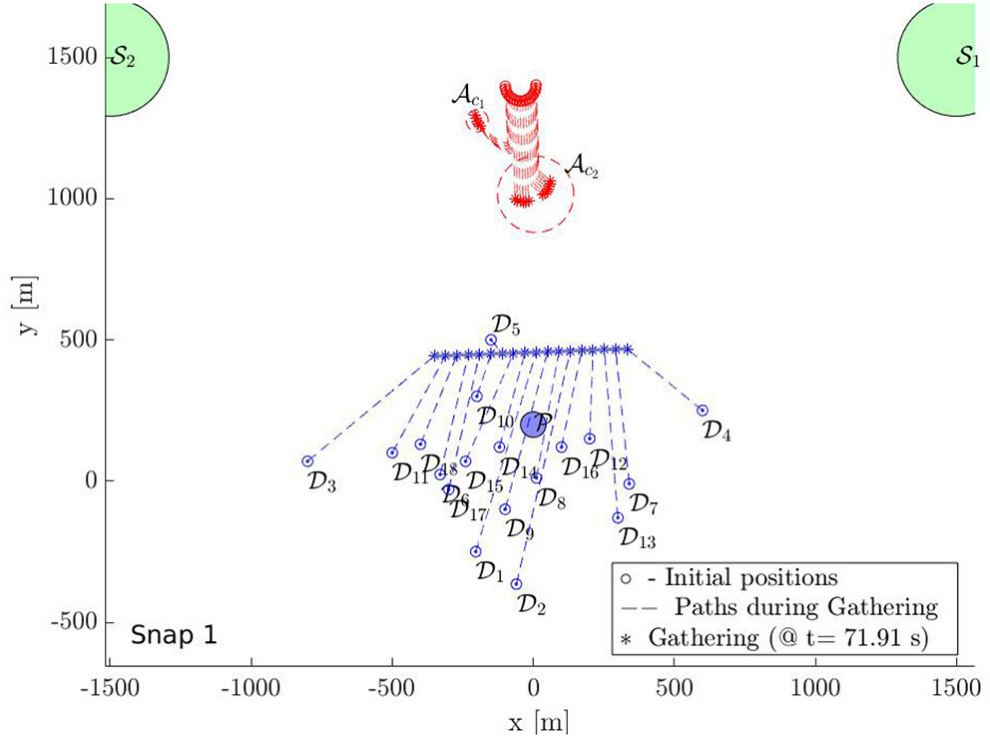
Multi-Swarm StringNet Herding: Snapshot 1.

**Figure 10:**
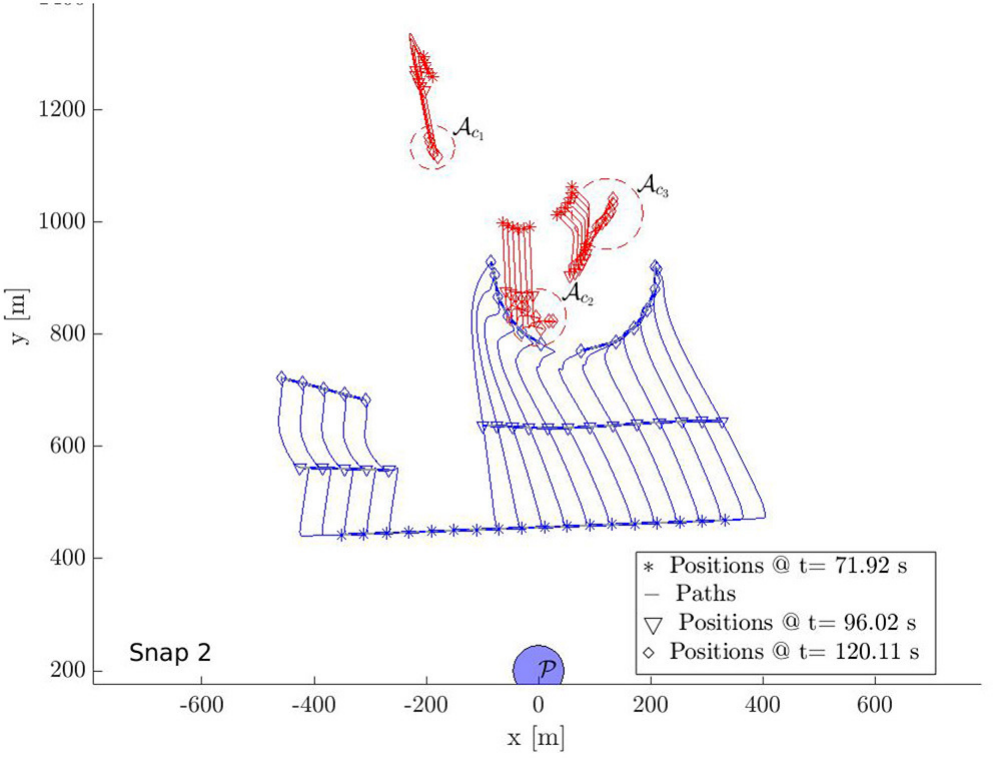
Multi-Swarm StringNet Herding: Snapshot 2.

**Figure 11:**
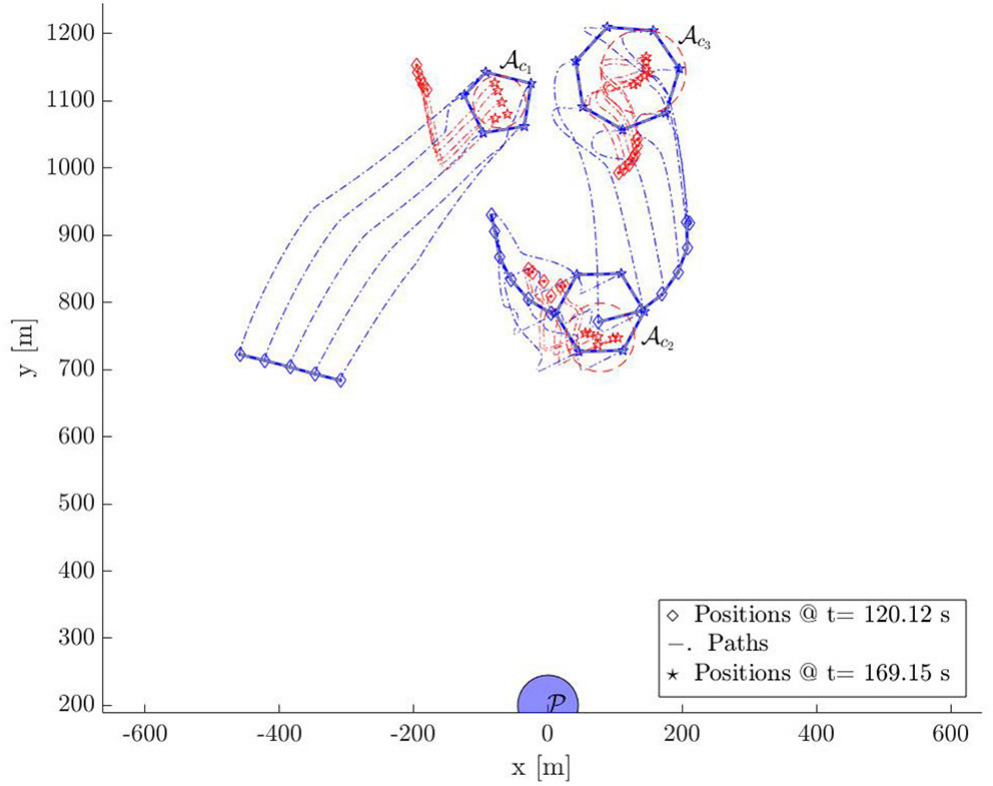
Multi-Swarm StringNet Herding: Snapshot 3.

**Figure 12:**
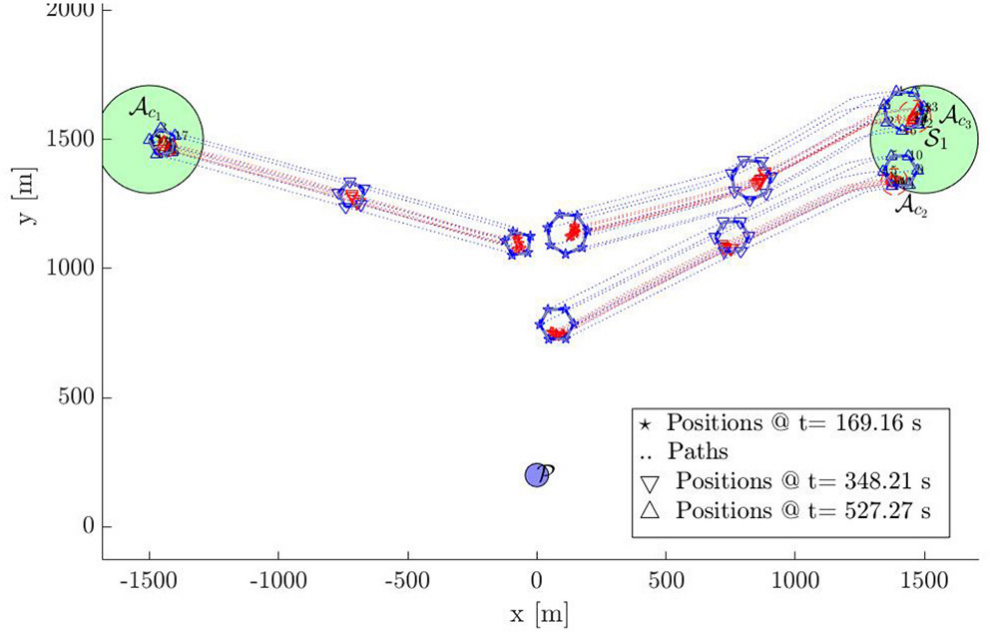
Multi-Swarm StringNet Herding: Snapshot 4.

In this section, we provide a simulation of 18 defenders herding 18 attackers to S with bounded control inputs. Figures 9–12 show the snapshots of the paths taken by all agents. The positions and paths of the defenders are shown in blue color, and that of the attackers in red. The string-barriers between the defenders are shown as wide solid blue lines with white dashes in them.

Snapshot 1 shows the paths during the gathering phase. As observed the defenders are able to gather at a location on the shortest path of the attackers to the protected area before the attacker reach there. Five attackers are already separated from the rest 13 in reaction to the incoming defenders in their path. The defenders have identified two swarms of the attackers $A_{c_{1}}$ and $A_{c_{2}}$ at the end of the gathering phase and assign two subgroups $D_{c_{1}}$ and $D_{c_{2}}$ of the defenders to $A_{c_{1}}$ and $A_{c_{2}}$ using Algorithm 1. As shown in snapshot 2, $D_{c_{1}}$ and $D_{c_{2}}$ seek $A_{c_{1}}$ and $A_{c_{2}}$, but the attackers in swarm $A_{c_{2}}$ further start splitting and the defenders identify this newly formed $A_{c_{2}}$ and $A_{c_{3}}$ at time *t* = 120.11 s. The group $D_{c_{2}}$ is then split into two subgroups $D_{c_{2}}$ and $D_{c_{3}}$ of appropriate sizes and assigned to the new swarms $A_{c_{2}}$ and $A_{c_{3}}$ using Algorithm 1.

Snapshot 3 shows how the 3 subgroups of the defenders are able to enclose the identified 3 swarms of the attackers by forming Closed-StringNets around them. Snapshot 4 shows how all the three enclosed swarms of the attackers are taken to the respective closest safe areas while each defenders' group ensures collision avoidance from other defenders' groups. Additional simulations can be found at https://tinyurl.com/yypb2yv9.

## 8. Experimental Results

**Figure 13:**
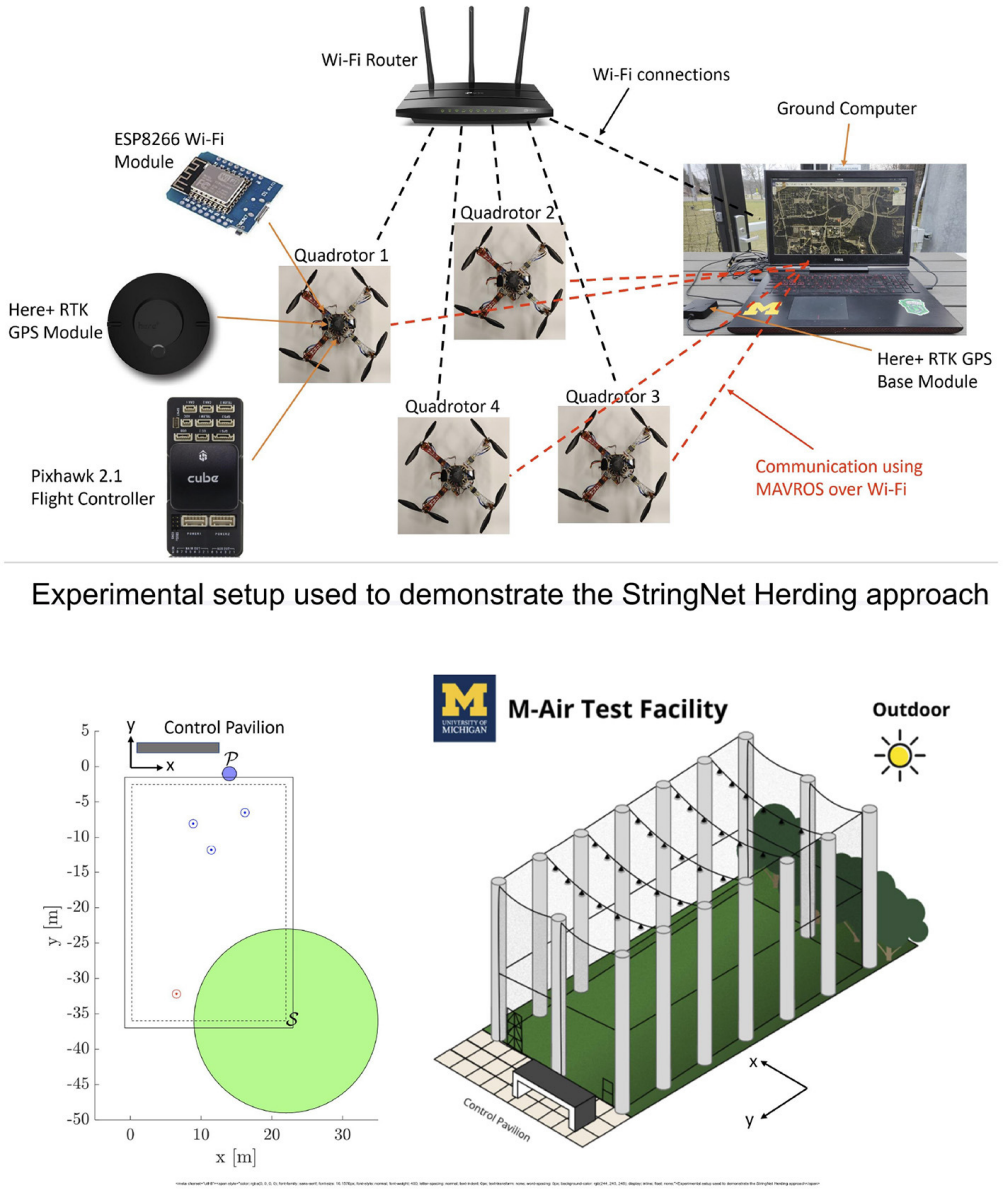
Experimental setup, M-Air facility and the initial configuration (The dotted line in the figure on the left shows the safe region of operation inside M-Air facility).

In this section, we provide hardware demonstrations of the herding approach. For this purpose, we use a fleet of in-house built 4 quadrotors each of which uses PixHawk cube 2.1 autopilot board for autonomous control. Each quadrotor is also fitted with Real-Time Kinetic (RTK) supported *here*+ GPS module and a ESP8266 Wi-Fi module. Figure 13 shows the overall experimental setup used for the demonstrations. The experimental setup consists of the fleet of quadrotors, a ground station computer, a wifi router, a RTK GPS base module. The ground station computer and the quadrotors are connected to a common wifi network created by the wifi router. The wifi modules on the quadrotors are used for the communication between the quadrotors and the ground station computer. The RTK GPS base module is used to provide corrections to the on-board GPS modules to provide centimeter level position accuracy. We use robot operating system (ROS) as an underlying framework to exchange and manipulate different signals used across the system. In particular, we use MAVROS package, based on MAVLink communication protocol, to exchange information between the ground station and the quadrotors.

As a proof of concept, we only demonstrate the “StringNet Herding” for single attacking swarm case in a centralized setting. In this setup, the ground station receives position and velocity commands from the vehicles and sends next reference commands, obtained through the MATLAB simulation running in the background based on the StringNet Herding formulation discussed earlier, to the quadrotors. The decentralized version for multi-swarm case can be tested similarly by having sufficient computational power available on the quadrotors.

We perform our experiments in an outdoor netted facility named M-Air at the University of Michigan. M-Air is a cuboid shaped netted facility as shown in Figure 13. We consider a scenario with 3 defenders $\left(D_{1},D_{2},D_{3}\right)$ and 1 attacker $\left(A_{1}\right)$ to demonstrate the proposed herding approach due to the limited space available in M-Air. The location of the protected area, the safe area and the initial locations of the quadrotors in M-Air are shown in Figure 13. We chose the protected area outside the M-Air so that we have large area available for quadrotors' motion. The safe area is chosen to be centered at one corner of M-Air with radius of 13 *m* so that the entire formation of the defenders after enclosing the attacker is able to reach inside the safe area.

**Figure 14:**
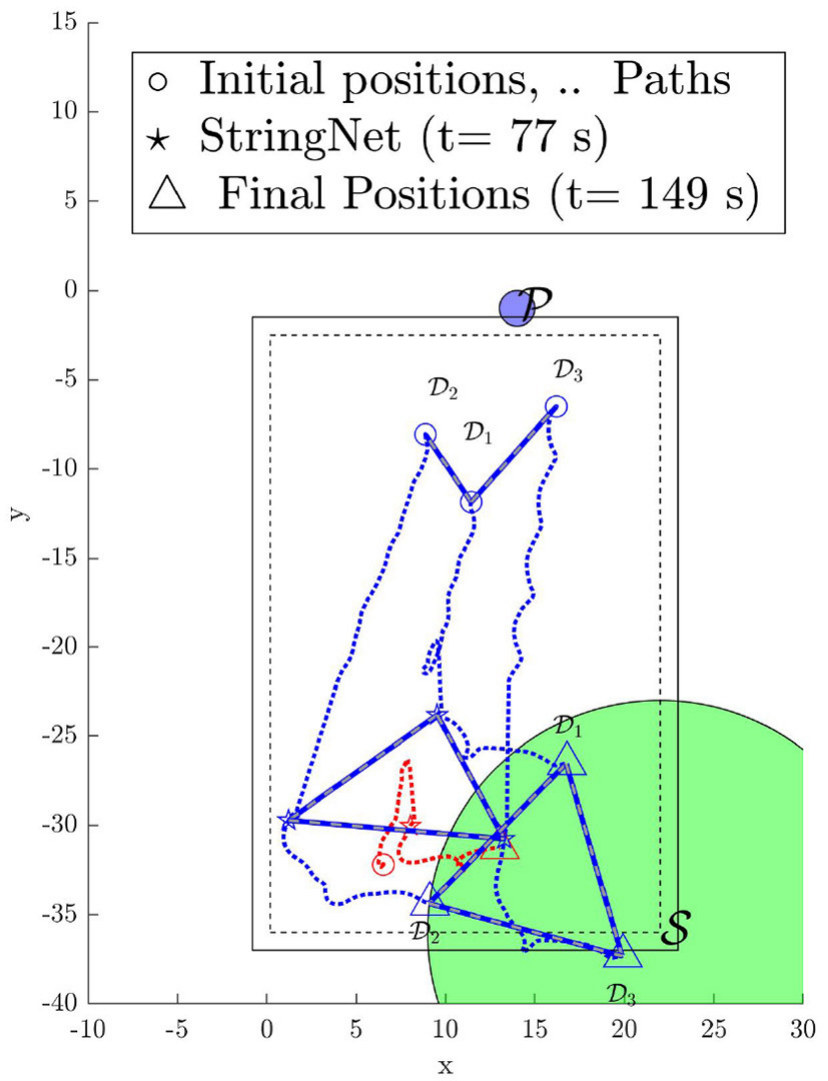
Paths traversed by the quadrotors during StringNet Herding (The dotted blue lines denote the paths of the defenders while the red denote that of the attackers. The solid blue lines with white dashes in them denote the string barrier assumed between the quadrotors).

**Figure 15:**
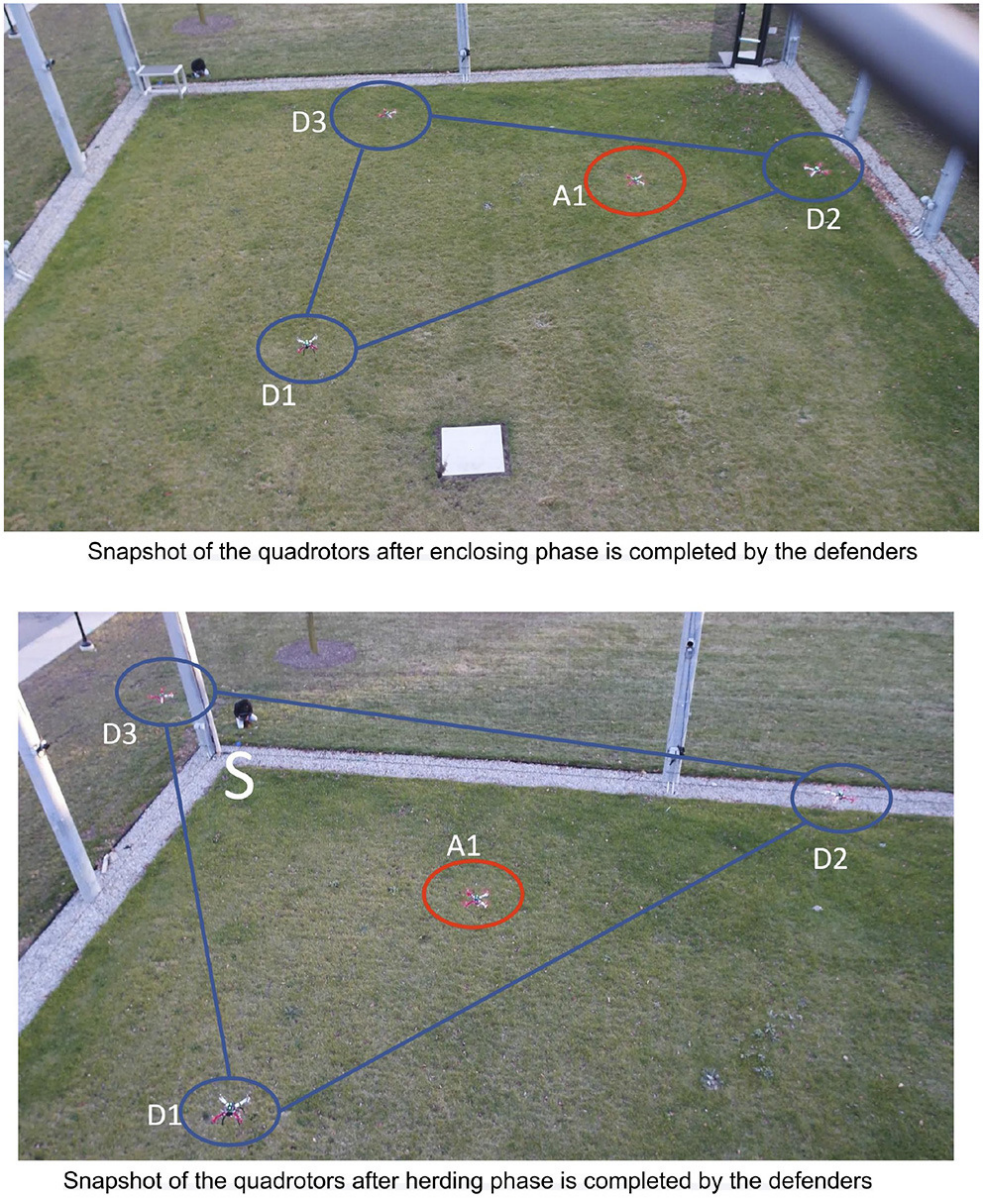
Snapshot of the quadrotors after enclosing and herding phases are completed by the defenders.

Again due to limited space, we only demonstrate the enclosing and the herding phases of the StringNet Herding approach. During the experiment all the quadrotors are commanded to fly at an altitude of 2.5 m above the local ground. The paths traversed by the quadrotors starting at the initial positions as shown in Figure 13 during the experiment are shown in Figure 14. The visuals of the quadrotors at different time instances during the experiment are shown in Figure 15. In Figure 15, the defenders $D_{1}$, $D_{2}$, and $D_{3}$ are denoted by D1, D2, and D3, respectively, with blue oval drawn around them to highlight where they are located in the figure. Similarly, the attacker is denoted by A1 and a red oval is drawn around it to highlight it.

As one can observe in Figure 14 the attacker starts moving toward the protected area in the beginning. Once it detects the defenders on its path, it starts to move away from them in order to protect itself. But, the defenders are able to enclose the attacker successfully at around *t* = 77 *s* despite attacker's initial attempt at escaping from them, see Figure 15 for the visual of the quadrotors at this instance. After the attacker is enclosed it is herded to the safe area located at the corner of M-Air at *t* = 149 *s* as shown in Figures 14, 15 and thus the protected area is protected from the attack by the attacker. The video of the experiment can be found at https://tinyurl.com/yyd3qfty.

## 9. Our Thoughts on Three Dimensional (3D) Case and Non-circular Geometries

The idea of StringNet can also be applied to 3D case. In our recent work Zhang et al. (2020), we extended the idea of “StringNet Herding” to the 3D case. The StringNet in 3D case, 3D-StringNet, is a single component, orientable triangle mesh with zero genus (holes) made of triangular net-like barrier faces. Similar to 2D-Stringnet herding, 3D-StringNet herding also consists of four phases: (1) gathering, (2) seeking, (3) enclosing and (4) herding. In Zhang et al. (2020), we design three 3D-StringNet formations of the defenders namely planar, hemispherical, spherical that are required to be achieved in the phases discussed above in order to effectively enclose the attackers and herd them to a safe area.

Although we assumed that the areas and the agents are circular, the proposed algorithm can be easily extended to non-circular geometries by considering appropriate distance metric in the algorithmic formulation.

## 10. Conclusions and Future Work

In this paper, we proposed a clustering-based, connectivity-constrained, assignment algorithm that distributes and assigns groups of defenders against swarms of the attackers, to herd them to the closest safe area using “StringNet Herding” approach. We provide two algorithms to solve this assignment problem: centralized and decentralized, and also a heuristic based on the optimal MIQCP that finds this assignment quickly. Furthermore, we provide conditions under which the defenders can successfully herd the attackers to safe areas.

Simulations show how this proposed multi-swarm herding method improves the original “StringNet Herding” method and enables the defenders herd all the attackers to safe areas even though the attackers start splitting into smaller swarms in reaction to the defenders. Hardware experiments demonstrate the success of the approach in real applications.

In our future work, we want to study a defense approach that combines herding and interception approach together in order to defend against wide range of attacks by the attackers.

## Data Availability Statement

The raw data supporting the conclusions of this article will be made available by the authors, without undue reservation.

## Author Contributions

VC and DP contributed to the conception and design of the study. VC formulated and simulated the theoretical algorithms and simulation case studies. VC and VM contributed to the development of experimental platform and experimental demonstrations of the herding algorithm. VC wrote major sections of the manuscript. DP is the principal investigator associated with this project. All authors contributed to manuscript revision, read, and approved the submitted version.

## Conflict of Interest

The authors declare that the research was conducted in the absence of any commercial or financial relationships that could be construed as a potential conflict of interest.

## Footnotes

^1^This is a better choice compared to a semicircular formation as chosen in Chipade and Panagou (2020b). Because, the semicircular formation, for a given length constraint on the string barrier (${\overset{-}{R}}_{sb}$), creates smaller blockage to the attackers as compared to the line formation. Although, Completing a circular formation starting from a semicircular formation of the same radius is faster. It is a trade-off between effectiveness and speed.

## 11. Appendix

### 11.1. Proof of Lemma 2

Sum of two convex functions is always a convex function (Boyd and Vandenberghe, 2004), so it is sufficient to show that ${\overset{-}{T}}_{d}\left(R\right)$ is a locally convex function to show that *f*(*R*) is a locally convex function. Let $g\left(R\right)={\overset{-}{T}}_{d}\left({\overset{-}{\varrho }}_{d}\left(R\right)\right)$. The double derivative of *g* is:

(14) $$\begin{array}{l} \begin{array}{c} \frac{\partial^{2}g}{\partial R^{2}}=\frac{\partial^{2}{\overset{-}{T}}_{d}}{\partial {{\overset{-}{\varrho }}_{d}}^{2}}{\left(\frac{\partial {\overset{-}{\varrho }}_{d}}{\partial R}\right)}^{2}+\frac{\partial {\overset{-}{T}}_{d}}{\partial {\overset{-}{\varrho }}_{d}}\frac{\partial^{2}{\overset{-}{\varrho }}_{d}}{\partial R^{2}}. \end{array} \end{array}$$

We have

(15a) $$\begin{array}{l} \frac{\partial {\overset{-}{T}}_{d}}{\partial {\overset{-}{\varrho }}_{d}}=\frac{C_{d}}{\lambda_{0}}\sqrt{\frac{\lambda +1}{\lambda -1}}\geq 0; \end{array}$$

(15b) $$\begin{array}{l} \frac{\partial^{2}{\overset{-}{T}}_{d}}{\partial {{\overset{-}{\varrho }}_{d}}^{2}}=\frac{1}{\lambda_{0}}\left(\frac{{\left(2C_{d}\right)}^{2}\lambda }{1-\lambda^{2}}\sqrt{\frac{\lambda +1}{\lambda -1}}\right)\leq 0; \end{array}$$

(15c) $$\begin{array}{l} \frac{\partial {\overset{-}{\varrho }}_{d}}{\partial R}=\sum_{j=1}^{N_{d}}\varrho_{j}^{\delta -2}{\left({\tilde{\varrho }}_{\delta }\right)}^{\frac{1}{\delta }-1}\left(R-R_{\theta j}\right); \end{array}$$

(15d) $$\begin{array}{l} \frac{\partial^{2}{\overset{-}{\varrho }}_{d}}{\partial R^{2}}=\sum_{j=1}^{N_{d}}{\left({\tilde{\varrho }}_{\delta }\right)}^{\frac{1}{\delta }-1}\varrho_{j}^{\delta -2}\{\left(\frac{1}{\delta }-1\right)\frac{\left(R-R_{\theta j}\right)}{\left({\tilde{\varrho }}_{\delta }\right)}\frac{\partial {\overset{-}{\varrho }}_{d}}{\partial R}\\ \;1+\left(\delta -2\right)\varrho_{j}^{-2}{\left(R-R_{\theta j}\right)}^{2}\}, \end{array}$$

where *R*_θ*j*_ = *R*_*j*_*cos*(|θ_*dj*_ − θ_*ac*_|). Let *R*^*^ be such that $\frac{\partial {\overset{-}{\varrho }}_{d}}{\partial R}{|}_{R=R^{*}}=0$. We have that ϱ_*j*_ is a convex function of *R* which implies that its ℓ_δ_-norm, ${\tilde{\varrho }}_{\delta }$, is also a convex function (Boyd and Vandenberghe, 2004). This means ${\tilde{\varrho }}_{\delta }\left(R^{*}\right)$ is the minimum value of ${\tilde{\varrho }}_{\delta }$, i.e., ${\tilde{\varrho }}_{\delta }\geq {\tilde{\varrho }}_{\delta }\left(R^{*}\right)$. Since not all defenders are co-located ${\tilde{\varrho }}_{\delta }\left(R^{*}\right)>0$ implying ${\tilde{\varrho }}_{\delta }>0$ and λ > 1. From Equation (15d), we have $\frac{\partial^{2}{\overset{-}{\varrho }}_{d}}{\partial R^{2}}{|}_{R=R^{*}}>0$. Then from Equation (14), we get $\frac{\partial^{2}g}{\partial R^{2}}{|}_{R=R^{*}}>0$. We know that ϱ_*j*_ is a twice continuously differentiable function of *R* for *R* > 0 and if we choose δ ≥ 2 then we can show that both $\frac{\partial {\overset{-}{\varrho }}_{d}}{\partial R}$ and $\frac{\partial^{2}{\overset{-}{\varrho }}_{d}}{\partial R^{2}}$ are continuous functions of *R*. From Equations (15a) and (15b), we have that $\frac{\partial {\overset{-}{T}}_{d}}{\partial {\overset{-}{\varrho }}_{d}}$ and $\frac{\partial^{2}{\overset{-}{T}}_{d}}{\partial {{\overset{-}{\varrho }}_{d}}^{2}}$ are continuous functions of *R*. This implies that $\frac{\partial^{2}g}{\partial R^{2}}$ is continuous at *R* = *R*^*^.

Combining the two results that $\frac{\partial^{2}g}{\partial R^{2}}$ is continuous and greater than 0 at *R* = *R*^*^ implies that there exists ϵ > 0 such that $\frac{\partial^{2}g}{\partial R^{2}}>0$ for all *R* satisfying |*R* − *R*^*^| < ϵ, i.e., *g*(*R*) is locally convex in the neighborhood of *R* = *R*^*^. Hence *f*(*R*) is also a locally convex function in the neighborhood of *R* = *R*^*^.

## References

[B1] Ankerst M. Breunig M. M. Kriegel H.-P. Sander J. (1999). Optics: ordering points to identify the clustering structure. ACM Sigmod Rec. 28, 49–60. 10.1145/304181.304187

[B2] Boyd S. Vandenberghe L. (2004). Convex Optimization. Cambridge: Cambridge University Press.

[B3] Burkard R. Dell'Amico M. Martello S. (2012). Assignment Problems, Revised Reprint, Vol. 106. Philadelphia, PA: Siam.

[B4] Cai N. Diao C. Khan M. J. (2017). A novel clustering method based on quasi-consensus motions of dynamical multiagent systems. Complexity 2017:4978613. 10.1155/2017/4978613

[B5] Chen M. Zhou Z. Tomlin C. J. (2017). Multiplayer reach-avoid games via pairwise outcomes. IEEE Trans. Autom. Control 62, 1451–1457. 10.1109/TAC.2016.2577619

[B6] Chipade V. S. Panagou D. (2019). Herding an adversarial swarm in an obstacle environment, in 2019 IEEE 58th Conference on Decision and Control (CDC) (Nice: IEEE), 3685–3690.

[B7] Chipade V. S. Panagou D. (2020a). Approximate time-optimal trajectories for damped double integrator in 2d obstacle environments under bounded inputs. arXiv [Preprints] arXiv:2007.

[B8] Chipade V. S. Panagou D. (2020b). Multi-agent planning and control for swarm herding in 2d obstacle environments under bounded inputs. IEEE Trans. Robot. Available online at: https://tinyurl.com/yy5k6943

[B9] Chipade V. S. Panagou D. (2020c). Multi-swarm herding: Protecting against adversarial swarms, in 2020 59th IEEE Conference on Decision and Control (CDC) (IEEE), 5374–5379. 10.1109/CDC42340.2020.9303837

[B10] Coon M. Panagou D. (2017). Control strategies for multiplayer target-attacker-defender differential games with double integrator dynamics, in Conference on Decision and Control (Melbourne, VIC: IEEE), 1496–1502.

[B11] Dai B. Li W. (2014). Flocking of -agents with arbitrary shape obstacle, in Proceedings of the 33rd Chinese Control Conference (Nanjing: IEEE), 1311–1316.

[B12] Deptula P. Bell Z. I. Zegers F. M. Licitra R. A. Dixon W. E. (2018). Single agent indirect herding via approximate dynamic programming, in 2018 IEEE Conference on Decision and Control (CDC) (Miami Beach, FL: IEEE), 7136–7141.

[B13] Ester M. Kriegel H.-P. Sander J. Xu X. (1996). A density-based algorithm for discovering clusters in large spatial databases with noise, in Kdd (Portland, OR), Vol. 96-34, 226–231.

[B14] Goel R. Lewis J. Goodrich M. Sujit P. (2019). Leader and16 predator based swarm steering for multiple tasks, in 2019 IEEE International Conference on Systems, Man and Cybernetics (SMC) (Bari: IEEE), 3791–3798.

[B15] Gurobi Optimization L. (2018). Gurobi Optimizer Reference Manual.

[B16] Haque M. A. Rahmani A. R. Egerstedt M. B. (2011). Biologically inspired confinement of multi-robot systems. Int. J. Bio Inspir. Comput. 3, 213–224. 10.1504/IJBIC.2011.041145

[B17] Kline A. Ahner D. Hill R. (2019). The weapon-target assignment problem. Comput. Operat. Res. 105, 226–236. 10.1016/j.cor.2018.10.015

[B18] Kuhn H. W. (1955). The hungarian method for the assignment problem. Naval Res. Log. Q. 2, 83–97. 10.1002/nav.3800020109

[B19] Licitra R. A. Bell Z. I. Doucette E. A. Dixon W. E. (2018). Single agent indirect herding of multiple targets: a switched adaptive control approach. IEEE Control Syst. Lett. 2, 127–132. 10.1109/LCSYS.2017.2763968

[B20] Licitra R. A. Hutcheson Z. D. Doucette E. A. Dixon W. E. (2017). Single agent herding of n-agents: a switched systems approach. IFAC PapersOnLine 50, 14374–14379. 10.1016/j.ifacol.2017.08.2020

[B21] MacQueen J. Le Cam L. M. Neyman J. (1967). Some methods for classification and analysis of multivariate observations, in Proceedings of the Fifth Berkeley Symposium on Mathematical Statistics and Probability, Vol. 1–14 (Oakland, CA), 281–297.

[B22] Mirjan A. Federico A. Raffaello D. Fabio G. Matthias K. (2016). Building a bridge with flying robots, in Robotic Fabrication in Architecture, Art and Design 2016 (Cham: Springer), 34–47.

[B23] Nardi S. Mazzitelli F. Pallottino L. (2018). A game theoretic robotic team coordination protocol for intruder herding. IEEE Robot. Autom. Lett. 3, 4124–4131. 10.1109/LRA.2018.2857004

[B24] O'Callaghan L. Mishra N. Meyerson A. Guha S. Motwani R. (2002). Streaming-data algorithms for high-quality clustering, in Proceedings 18th International Conference on Data Engineering (San Jose, CA: IEEE), 685–694.

[B25] Öncan T. (2007). A survey of the generalized assignment problem and its applications. Inform. Syst. Oper. Res. 45, 123–141. 10.3138/infor.45.3.123

[B26] Paranjape A. A. Chung S.-J. Kim K. Shim D. H. (2018). Robotic herding of a flock of birds using an unmanned aerial vehicle. IEEE Trans. Robot. 34, 901–915. 10.1109/TRO.2018.2853610

[B27] Pierson A. Schwager M. (2018). Controlling noncooperative herds with robotic herders. IEEE Trans. Robot. 34, 517–525. 10.1109/TRO.2017.2776308

[B28] Raghuwaiya K. Vanualailai J. Sharma B. (2016). Formation splitting and merging, in International Conference on Swarm Intelligence (Bali: Springer), 461–469.

[B29] Rezende M. D. De Lima B. S. P. Guimarães S. (2018). A greedy ant colony system for defensive resource assignment problems. Appl. Artif. Intell. 32, 138–152. 10.1080/08839514.2018.1451137

[B30] Sharan R. Shamir R. (2000). Click: a clustering algorithm with applications to gene expression analysis. Proc. Int. Conf. Intell. Syst. Mol. Biol. 8, 307–316. 10977092

[B31] Shishika D. Paulos J. Kumar V. (2020). Cooperative team strategies for multi-player perimeter-defense games. IEEE Robot. Autom. Lett. 5, 2738–2745. 10.1109/LRA.2020.2972818

[B32] Stipanović D. M. Tomlin C. J. Leitmann G. (2012). Monotone approximations of minimum and maximum functions and multi-objective problems. Appl. Math. Optimiz. 66, 455–473. 10.1007/s00245-012-9179-8

[B33] Varava A. Hang K. Kragic D. Pokorny F. T. (2017). Herding by caging: a topological approach towards guiding moving agents via mobile robots, in Proceedings of Robotics: Science and Systems (Cambridge, MA). 10.15607/RSS.2017.XIII.074

[B34] Xu D. Tian Y. (2015). A comprehensive survey of clustering algorithms. Ann. Data Sci. 2, 165–193. 10.1007/s40745-015-0040-1

[B35] Yan R. Shi Z. Zhong Y. (2019). Task assignment for multiplayer reach–avoid games in convex domains via analytical barriers. IEEE Trans. Robot. 36, 107–124. 10.1109/TRO.2019.2935345

[B36] Zhang T. Ramakrishnan R. Livny M. (1996). Birch: an efficient data clustering method for very large databases. ACM Sigmod Rec. 25, 103–114. 10.1145/235968.233324

[B37] Zhang W. Chipade V. S. Panagou D. (2020). Herding an adversarial swarm in three-dimensional spaces. arXiv preprint arXiv:2007.04406.

